# Distribution and Characterisation of Tick-Borne Flavi-, Flavi-like, and Phenuiviruses in the Chelyabinsk Region of Russia

**DOI:** 10.3390/v14122699

**Published:** 2022-12-01

**Authors:** Ivan S. Kholodilov, Oxana A. Belova, Anna Y. Ivannikova, Magomed N. Gadzhikurbanov, Marat T. Makenov, Alexander S. Yakovlev, Alexandra E. Polienko, Alena V. Dereventsova, Alexander G. Litov, Larissa V. Gmyl, Egor V. Okhezin, Svetlana V. Luchinina, Alexander S. Klimentov, Galina G. Karganova

**Affiliations:** 1 Laboratory of Biology of Arboviruses, FSASI Chumakov Federal Scientific Center for Research and Development of Immune-and-Biological Products of RAS, 108819 Moscow, Russia; 2 Department of Biology, Lomonosov Moscow State University, 119234 Moscow, Russia; 3 Department of Molecular Diagnostics and Epidemiology, Central Research Institute of Epidemiology, 111123 Moscow, Russia; 4 Laboratory of Biochemistry, FSASI Chumakov Federal Scientific Center for Research and Development of Immune-and-Biological Products of RAS, 108819 Moscow, Russia; 5 Office of Rospotrebnadzor in the Chelyabinsk Region, 454092 Chelyabinsk, Russia

**Keywords:** Alongshan virus, Yanggou tick virus, Jingmenvirus group, tick-borne encephalitis virus, *Flavivirus*, flavi-like virus, *Phlebovirus*, segmented virus, tick cell line, combined foci

## Abstract

In this work, we presented data from a two-year study of flavi-, flavi-like, and phenuiviruses circulation in the population of ixodid ticks in the Chelyabinsk region. We isolated three tick-borne encephalitis virus (TBEV) strains from *I. persulcatus*, which was not detected in the ticks of the genus *Dermacentor*. The virus prevalence ranged from 0.66% to 2.28%. The Yanggou tick virus (YGTV) is widespread in steppe and forest-steppe zones and is mainly associated with ticks of the genus *Dermacentor*. We isolated 26 strains from *D. reticulatus*, *D. marginatus*, and *I. persulcatus* ticks in the HAE/CTVM8 tick cell line. The virus prevalence ranged from 1.58% to 4.18% in *D. reticulatus*, ranged from 0.78% to 3.93% in *D. marginatus*, and was 0.66% in *I. persulcatus*. There was combined focus of TBEV and YGTV in the territory of the Chelyabinsk region. The Alongshan virus (ALSV) was found to be associated with *I. persulcatus* ticks and is spread in forest zone. We detected 12 amplicons and isolated 7 strains of ALSV in tick cells. The virus prevalence ranged from 1.13% to 6.00%. The phlebovirus Gomselga and unclassified phenuivirus Stavropol were associated with *I. persulcatus* and *D. reticulatus* ticks, respectively. Virus prevalence of the unclassified phenuivirus Stavropol in the Chelyabinsk region is lower than that in neighbouring regions.

## 1. Introduction

In recent decades, a large number of arthropod-borne viruses have been discovered, and their number is growing every year [1,2,3,4,5]. Some of them may be pathogenic for mammals, including humans [3,6,7,8]; the role of others as pathogens has not been established and requires study to understand their pathogenic potential and impact on the epidemiology and epizootology of known pathogens.

The genus *Flavivirus* belongs to the family *Flaviviridae*, and includes small enveloped viruses with a non-segmented ssRNA(+) genome of about 10 kb in length that encodes one large open reading frame, which codes a single polyprotein that is co- and post-translationally cleaved by viral and cellular proteases into three structural (C, prM, and E) and seven non-structural (NS1, NS2A, NS2B, NS3, NS4A, NS4B, and NS5) proteins [9]. The representatives of the genus *Flavivirus* are the cause of severe human diseases with damage to the central nervous system and haemorrhagic fevers [10,11,12,13,14]. Currently, both mosquito-borne viruses (West Nile virus [15,16] and Japanese encephalitis virus [17]) and tick-borne viruses (tick-borne encephalitis virus (TBEV) [11], Omsk haemorrhagic fever virus [10,18], Powassan virus [19], Louping ill virus [20]) are circulating in Russia.

In the last decade, flavi-like viruses with a segmented genome have been discovered [4], and these viruses were included in the group of unclassified viruses related to the family *Flaviviridae* [9]. Segmented flavi-like viruses, such as the Jingmen tick virus (JMTV), Alongshan virus (ALSV), Yanggou tick virus (YGTV), and others, have a segmented ssRNA(+) genome, two segments of which have homology with the well-studied flavivirus RNA-dependent RNA polymerase NS5 and RNA helicase-protease NS3, with the remaining two segments being specific to these viruses [4,21]. It has recently been shown that ALSV VP1a shares 23% identical amino acids and 50% chemically similar amino acids with the yellow fever virus envelope (E) protein over a 190 amino acid length segment [22]. Representatives of this group were detected in different arthropods [1,4,21,23,24,25,26,27], reptiles [28], and mammals [24,27,29,30,31], including humans [6,7,32]. There is a wide circulation of segmented flavi-like viruses, such as ALSV, YGTV, and JMTV, in Russia [23,33,34].

The recently updated classification of the family *Phenuiviridae* includes 20 quite heterogeneous genera [35]. The genome of phenuiviruses is represented by three segments of negative or ambisense ssRNA: the large (L) segment encodes RNA-dependent RNA polymerase; the medium (M) segment encodes glycoproteins (Gn and Gc) and, in some viruses, an accessory non-structural NSm protein; and the small (S) segment encodes a nucleoprotein (N) and non-structural NSs protein [36]. Viruses are hosted by blood-feeding arthropods, such as ticks, mosquitoes, and sandflies. Some of the phenuiviruses belong to the ecological group of arboviruses, are transmitted between mammals through the bites of blood-feeding arthropods, and are associated with human diseases [2,3,8]. Previously, we showed a wide distribution and diversity of representatives of the genera *Phlebovirus*, *Ixovirus*, and unclassified phenuiviruses in Russia [37,38].

Combined foci of the tick-borne pathogens, which are inhabited by different species of ticks, attract much attention [39,40]. A comprehensive assessment of such foci provides an idea of the functioning of the virus–tick–host system and is necessary for understanding the evolution of ecological systems.

The Chelyabinsk region is one of such focus. The region is located in the south of the central part of Russia and borders with Kazakhstan. Three natural zones are distinguished on the Chelyabinsk region territory: mountain forest (mountain taiga, coniferous, deciduous, and mixed forests), forest-steppe, and steppe [41]. The region is inhabited by various species of ticks: *Ixodes persulcatus, Ixodes trianguliceps*, *Ixodes crenulatus*, *Ixodes apronophorus*, *Dermacentor reticulatus*, and *Dermacentor marginatus* [23,42,43,44], but in our study we considered the most common pasture-questing tick species (nemobionts [45]). The mountain forest zone inhabits only *I. persulcatus* ticks; forest-steppe zone inhabits *D. reticulatus* and *I. persulcatus* ticks in approximately equal proportions; and the steppe zone inhabits mainly *D. marginatus* and *D. reticulatus* ticks, as well as a small number of *I. persulcatus* ticks [43].

TBEV is one of the main arboviruses in Russia, being the cause of severe human diseases [11]. The entire territory of the Chelyabinsk region is endemic for tick-borne encephalitis (TBE) [11,44]. TBE incidence is registered in the whole region, but TBEV has been detected in ticks only in mountain forest and forest-steppe zones, where *I. persulcatus* ticks are located [43,44,46,47].

Previously, we tested ticks collected in the Chelyabinsk region in 2014 using primers for the genus *Flavivirus* [33]. We detected nine positive samples. The nucleotide sequence of the PCR product showed that it was ALSV. In ticks collected in 2015 in the same place that ALSV was previously found, we detected two positive samples using ALSV-specific primers. We isolated four strains from the ticks collected in 2014 and two strains from the ticks collected in 2015 in the IRE/CTVM19 tick cell line [23,33].

In this work, we tested previously unstudied ticks collected in 2015 using pan-flavi and ALSV-specific primers, as well as studying all ticks collected in 2014 and 2015 for the presence of the representatives of the genus *Phenuivirus*, YGTV, and TBEV. We provide newly obtained data from a two-year study of the circulation of flavi-, flavi-like, and phenuiviruses in the population of ixodid ticks in the Chelyabinsk region.

## 2. Materials and Methods

### 2.1. Collection and Processing of Ticks

**Figure 1:**
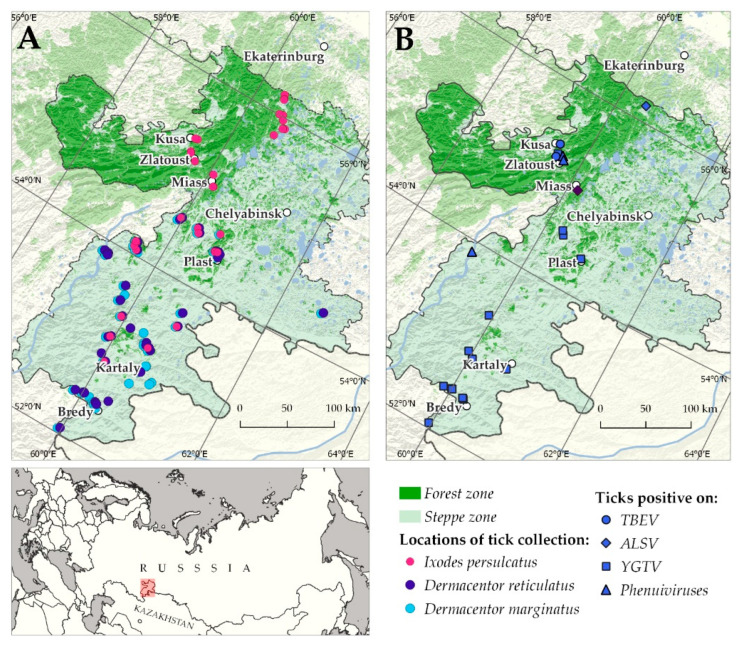
The map of the Chelyabinsk region. (**A**). Locations of tick collections in the Chelyabinsk region. (**B**). Locations of detection of TBEV, Yanggou tick virus, Alongshan virus, and phenuiviruses in the Chelyabinsk region. ALSV near city Miass (purple rhombus, **B**) was described previously [23,33].

Ticks were collected by flagging from vegetation in the Chelyabinsk region in 2014 and 2015 at the end of May (Figure 1). Ticks were identified using taxonomic keys [48,49]. The locations of tick collections and tick species are presented (Figure 1A and Table S1). In Table S1, ticks, which were previously tested for the presence of flaviviruses and ALSV, were highlighted in grey.

Adult ticks were homogenised in pools (two to six individuals) according to species composition, location, and site of collection using the laboratory homogeniser TissueLyser II (QIAGEN, Germany) in 0.9% saline solution (FSASI Chumakov FSC R&D IBP RAS, Moscow, Russia). The volume of the solution added was dependent on the tick’s species and the number of ticks in the pool: for each *Ixodes* sp. tick, 150 µL of solution was added; for each *Dermacentor* spp. tick, 200 µL of solution was added.

### 2.2. Infection of Tick Cell Line

We used a cell line derived from embryos of the ticks *Hyalomma anatolicum* (HAE/CTVM8) [50] provided by the Tick Cell Biobank (Liverpool, UK). The tick cell line was maintained at 28 °C in L-15 (Leibovitz) medium (FSASI Chumakov FSC R&D IBP RAS, Moscow, Russia) supplemented with 10% tryptose phosphate broth (Difco, Detroit, MI, USA), 20% foetal bovine serum (Gibco, Invitrogen, Carlsbad, CA, USA), 2 mML-glutamine, and antibiotics, as described previously [23]. Infection of cells and harvest of the virus were carried out, as described previously [23]. Prior to infection, HAE/CTVM8 cells were seeded in flat-sided culture tubes (Nunc, ThermoFisher Scientific, Waltham, MA, USA) in 2.2 mL of complete medium and incubated at 28 °C. A week later, cells were infected by adding 200 µL of unfiltered tick homogenate and incubated at 28 °C. Medium was changed at weekly intervals by removal and replacement of 1.1 mL; the spent medium was used to harvest the virus. Once every 1–2 months we checked the culture medium by RT-PCR for presence of the virus using specific primers.

### 2.3. Infection of Mammalian Cell Line

One-day-old culture of pig embryo kidney (PEK) cells was infected with tick suspensions that were PCR-positive for the presence of TBEV RNA, as described previously [51]. One hundred microliters of tick suspension was added to each well of a 24-well panel (24-well cell culture plate, Costar) with the cell culture and incubated for 1 h at 37 °C. Then, 1 mL of a maintenance medium consisting of medium 199 (FSASI Chumakov FSC R&D IBP RAS, Moscow, Russia), 2% bovine serum (Gibco, Invitrogen, Carlsbad, CA, USA), and antibiotics (100 U/mL penicillin, 100 μg/mL streptomycin) were added, and the mixture was incubated at 37 °C for 7 days. The infected culture supernate was harvested immediately after the appearance of the cytopathogenic effect (CPE) or on the seventh day after infection in the absence of CPE.

### 2.4. Reverse-Transcriptase PCR (RT-PCR) and Sequencing of Amplified Products

Viral RNA from tick suspensions and infected cell culture supernate was isolated with TRI Reagent LS (Sigma-Aldrich, St. Louis, MO, USA), according to the manufacturer’s protocols. Reverse transcription was performed with random hexamer primer (R6) and M-MLV reverse transcriptase (Promega, Madison, WI, USA), according to the manufacturer’s protocols. To detect the virus, newly obtained viral genomic cDNA was amplified by PCR using primers for the genus *Flavivirus* [52], and specific primers for ALSV: Miass_gly_3F and Miass_gly_3R [33]. In addition, previously described [23] and newly obtained viral genomic cDNA were amplified by PCR using pan-phlebovirus primers PhlP2 and PhlM2 [53]; specific primers for YGTV: Yanggou_gly_1F and Yanggou_gly_1R (Table S2); and for TBEV: Kgg19 and Kgg31 [54]. To sequence the complete or partial genome, viral genomic cDNA was amplified by PCR using specific primers for ALSV [23], YGTV (Table S2), TBEV [51], and phenuiviruses [53]. The PCR product was gel-purified and then sequenced in both directions on the ABI PRISM 3500 (Applied Biosystems, Foster City, CA, USA) sequencer using ABI PRISM^®^ BigDye™ Terminator v. 3.1. Genomic sequences were assembled using SeqMan software (DNAstar, Madison, WI, USA).

### 2.5. Phylogenetic Analysis

#### 2.5.1. Tick-Borne Encephalitis Virus

RNA sequences of all published TBEV strains from Chelyabinsk region, some representatives of all TBEV subtypes, and the strains described in this article were used in the phylogenetic analysis. The nucleotide sequences of the genome-coding region of protein E (352 bp) were aligned using ClustalW. Phylogenetic analysis was conducted using the maximum likelihood method and the Tamura–Nei model [55] in MEGA X with 1000 bootstrap replications [56].

#### 2.5.2. Flavi-like Viruses

RNA sequences of all published strains of YGTV, representatives of JMTV and ALSV, and all strains described in this article were used in the phylogenetic analysis. The nucleotide sequences of the genome-coding regions of segment 2 were aligned using ClustalW. Phylogenetic analysis of the fragments of segment 2 (296 bp) was conducted using the maximum likelihood method and the Tamura–Nei model [55] in MEGA X with 1000 bootstrap replications [56].

#### 2.5.3. Phenuiviruses

RNA sequences of some representatives of the family *Phenuiviridae* and amplicons described in this article were used in the phylogenetic analysis. The nucleotide sequences of the genome-coding regions of segment L were aligned using ClustalW. Phylogenetic analysis of the fragments of L segments (474 bp) was conducted using the maximum likelihood method and the Tamura–Nei model [55] in MEGA X with 1000 bootstrap replications [56].

### 2.6. Data Analysis

For pooled samples with variable pool sizes, we calculated the prevalence and confidence intervals using a maximum likelihood estimator with the assumption of 100% test sensitivity and specificity [57]. The calculations were performed on the EPITOOLS web platform (https://epitools.ausvet.com.au) (accessed on 2 June 2022). Comparison of data sets was carried out using the Wilcoxon–Mann–Whitney test in R [58]. Graphs and diagrams were also plotted in R. Maps were drawn in QGIS software [59].

To identify the open reading frames in segment 2 of ALSV and YGTV, we used the Snap Gene Viewer program with translation options: minimum length 75 amino acids, selected options “Require a start codon ATG”, “except at DNA ends”, and “Standard” of genetic code for ORFs and new features. Percent identity of nucleotide and amino acid sequences of TBEV, Yanggou tick virus, and Alongshan virus were computed in MEGA X program using the “Compute pairwise distance” function with default settings [56].

## 3. Results

We collected the samples in 2014 and 2015, and studied 3960 ticks (*I. persulcatus*—1275, *D. reticulatus*—1063, *D. marginatus*—1622). The locations of the tick collection and tick species are presented in Figure 1A.

Two cohorts of ticks were used in the study. The first one, collected in 2014 and 2015 in the amount of 1958 individuals (*I. persulcatus*—402, *D. reticulatus*—594, *D. marginatus*—962), was previously tested for the presence of flaviviruses using panflavi primers and ALSV using specific primers [23]. The second one was collected in 2015 (*I. persulcatus*—873, *D. reticulatus*—469, *D. marginatus*—660; in total—2002 individuals) and it was unstudied prior to this work. In this work, the first cohort was additionally tested for the presence of TBEV, YGTV using specific primers, and phenuiviruses using pan-phlebovirus primers. The second cohort was tested using all of the abovementioned primer sets (pan-flavi, pan-phlebo, and specific for TBEV, ALSV, and YGTV).

We did not find West Nile virus, Japanese encephalitis virus, Omsk haemorrhagic fever virus, Powassan virus, or Louping ill virus in ticks in the Chelyabinsk region using primers for the genus *Flavivirus*.

### 3.1. Tick-Borne Encephalitis Virus

No TBEV RNA was detected using either pan-flavi primers or TBEV-specific primers in the first tick cohort. TBEV RNA was detected in unfed *I. persulcatus* ticks that were collected in 2015 (second cohort) in forest area near cities Zlatoust (strains Zlatoust15-T22241 and Zlatoust15-T22637) and Kusa (strain Kusa15-T22532) (Figure 1B) using primers specific for TBEV and for the genus *Flavivirus*. All strains were isolated from ticks in the PEK cell line. TBEV RNA was not found in any tick of the genus *Dermacentor*. The virus prevalence in *I. persulcatus* ranged from 0.66% to 2.28% in different collection sites (Table 1) and was 0.24% in the whole region.

**Figure 2:**
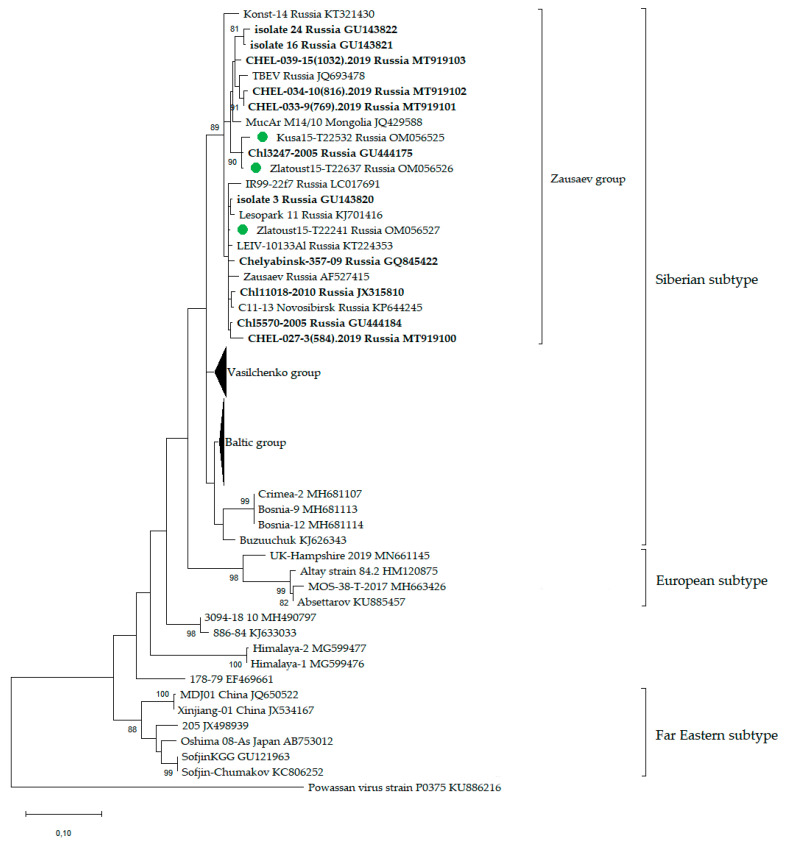
Phylogenetic analysis of the tick-borne encephalitis virus. Phylogenetic trees were constructed using E protein fragments (352 bp) in MEGA X with the maximum likelihood method (1000 bootstrap replications). Bootstrap values (>70%) are shown at the branches. GenBank accession numbers are listed for each strain. Green circles—strains of TBEV described in this study. All other TBEV strains from the Chelyabinsk region are in bold font.

The strain Kusa15-T22532 was found to be more similar to the strain Zlatoust15-T22637—the nucleotide identity was 99.12% and the amino acid identity was 99.60% in the genome fragment encoding protein E (Table S3). The distance between the collection points where these two strains were found was about 20 km. The third strain, Zlatoust15-T22241, was less similar to the strains Kusa15-T22532 and Zlatoust15-T22637 and had 96.31% and 96.81% nucleotide identities, respectively, in the genome fragment encoding protein E. According to the phylogenetic analysis, our strains Kusa15-T22532, Zlatoust15-T22637, and Zlatoust15-T22241 with other strains from the Chelyabinsk region and strains from Novosibirsk, Irkutsk, Altay, and Mongolia belonged to the Zausaev group of the Siberian subtype (Figure 2).

### 3.2. Segmented Flavi-like Viruses

#### 3.2.1. Alongshan Virus

The viral RNA of ALSV was detected only in *I. persulcatus* ticks collected in 2014 and 2015 in the forest area (Figure 1B, Table 1). Previously, we described the detection of nine ALSV-positive pools of *I. persulcatus* ticks collected in 2014 near the city Miass using primers for the genus *Flavivirus* and two ALSV-positive pools of *I. persulcatus* ticks collected in 2015 in the same collection site using specific primers [23,33]. In this study, we additionally detected and isolated one ALSV strain (Salma15-T22545) in *I. persulcatus* ticks from the second cohort using specific primers. This strain successfully persisted in HAE/CTVM8 cell lines for 6 months without a cytopathic effect. Overall virus prevalence between 2014–2015 ranged from 1.13% to 6.00%.

**Figure 3:**
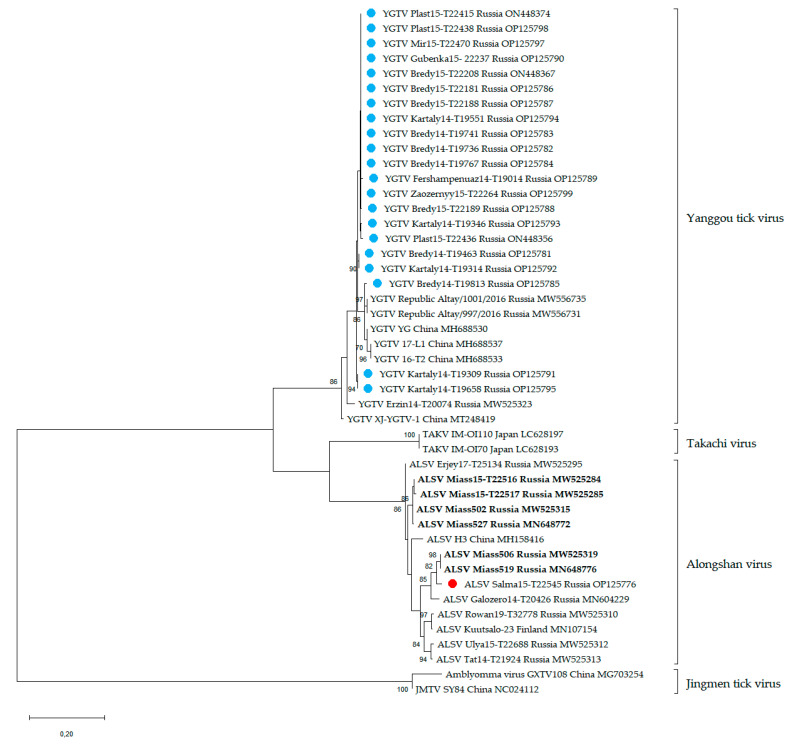
Phylogenetic analysis of flavi-like viruses. Phylogenetic trees were constructed using a fragment of segment 2 (296 bp) in MEGA X with the maximum likelihood method (1000 bootstrap replications). Bootstrap values (>70%) are shown at the branches. GenBank accession numbers are listed for each entry. Blue circles—strains of the Yanggou tick virus (YGTV) from the Chelyabinsk region described in this study. Red circles—strains of the Alongshan virus (ALSV) from the Chelyabinsk region described in this study. Bold font—ALSV strains from the Chelyabinsk region described previously.

For the phylogenetic analysis, we used all published homologous RNA sequences of ALSV, including strains described in this article. The phylogenetic analysis was performed on the fragment (296 bp) of segment 2 (Figure 3). All of our strains clustered with other strains of ALSV.

**Figure 4:**
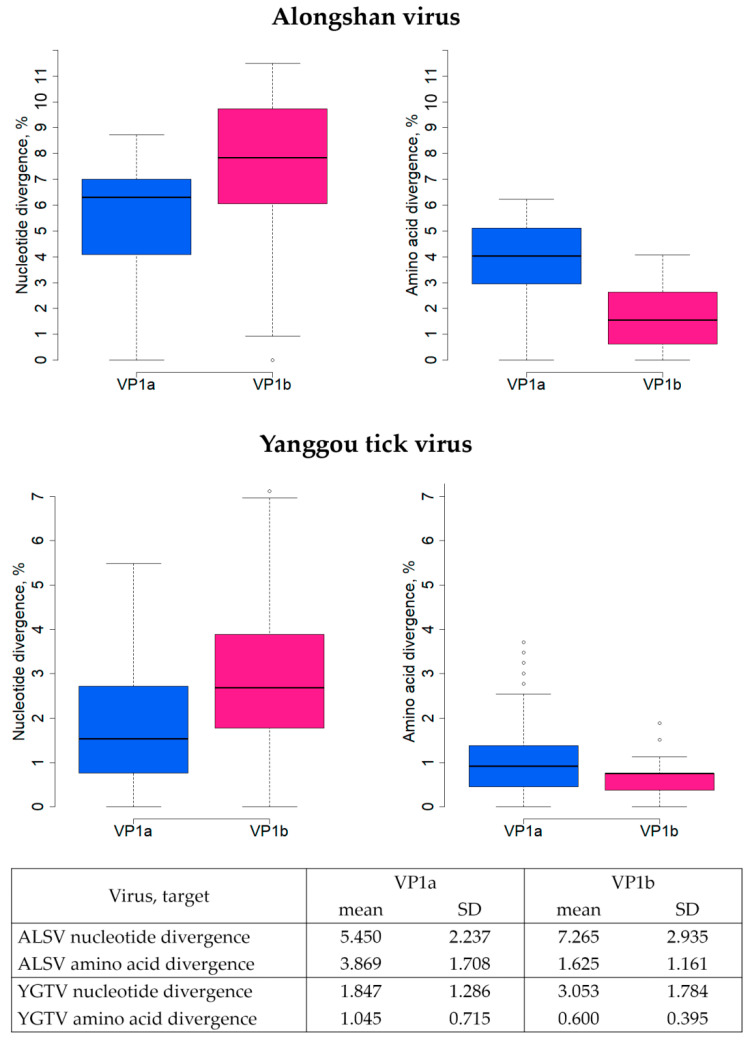
Estimates of evolutionary divergence between complete sequences of VP1a and VP1b proteins of the Yanggou tick virus and Alongshan virus. The table below represents mean and standard deviation (SD) of sequence divergences. The solid line represents the median, the box shows the interquartile range (IQR), and the whiskers represent 1.5 × IQR, the circles represent outliers (values exceeded 1.5 × IQR). Percentage identity of nucleotide and amino acid sequences of proteins VP1a and VP1b of each strain of the Yanggou tick virus and Alongshan virus were used to calculate the divergence (identity percentage) and are shown in Tables S4–S11. VP1a protein analysis includes 26 sequences of the Yanggou tick virus and 16 sequences of the Alongshan virus; VP1b protein analysis includes 16 sequences of the Yanggou tick virus and 16 sequences of the Alongshan virus.

Estimates of the evolutionary divergence between all ALSV sequences (from China, Russia, Finland, and France) of VP1a and VP1b proteins showed that greater divergence was observed in the nucleotide sequence of VP1b than of VP1a, and the average divergences were 7.27% and 5.45%, respectively (Wilcoxon–Mann–Whitney test *p* < 0.05). Greater divergence in amino acid sequences was observed in VP1a than in VP1b, and average divergences were 3.87% and 1.63%, respectively (Wilcoxon–Mann–Whitney test *p* < 0.05) (Figure 4).

#### 3.2.2. Yanggou Tick Virus

**Figure 5:**
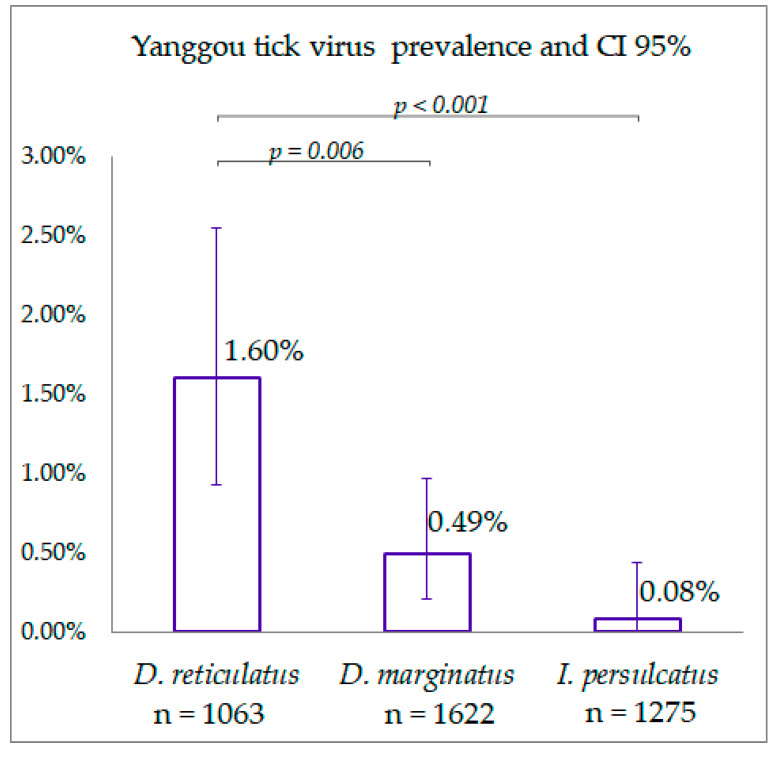
Mean Yanggou tick virus prevalence in different tick species calculated in all samples of the Chelyabinsk region. Error bars represent the 95% confidence intervals. The p-values of Fisher’s exact test are shown for species with significant differences in prevalence.

All ticks collected in 2014 and 2015 were tested for YGTV using specific primers. The viral RNA of YGTV was detected in 17 pools of *D. reticulatus*, in 8 pools of *D. marginatus* ticks collected in grassland areas, and only in 1 pool of *I. persulcatus* tick in forest area (Figure 1B, Table 1). All positive samples were detected using specific primers for YGTV. Moreover, all positive samples were detected in pools that were previously negative for the presence of flaviviruses. We estimated the prevalence and confidence intervals for all locations and tick species. However, some locations presented with a low number of ticks (less than 20), and in such cases, the estimated prevalence was not reliable. Therefore, we did not include the low-number locations in further analysis. The virus prevalence in different collection points ranged from 1.58% to 4.18% in *D. reticulatus* and from 0.78% to 3.93% in *D. marginatus*, and it was 0.66% in *I. persulcatus* ticks. The total prevalence of YGTV (calculated by all locations) in *D. reticulatus* was significantly higher than in *D. marginatus* and *I. persulcatus* (Fisher’s exact test *p* = 0.006 and, *p* < 0.001, respectively) (Figure 5). There was no significant difference in YGTV prevalence between *D. marginatus* and *I. persulcatus* (Fisher’s exact test *p* = 0.068) (Figure 5).

All strains of YGTV successfully persisted in the HAE/CTVM8 cell line for 10 months without cytopathic effect.

For the phylogenetic analysis, we used all published homologous RNA sequences of YGTV, including strains described in this article. The phylogenetic analysis was performed on the fragments (296 bp) of segment 2 (Figure 3). All our strains clustered with other strains of YGTV and formed one monophyletic group. When conducting phylogenetic analysis of the complete nucleotide and amino acid sequences of the proteins VP1a and VP1b, YGTV strains also formed one monophyletic group (Figures S1–S4).

The estimates of the evolutionary divergence between all complete YGTV sequences (from China and Russia) of the VP1a and VP1b proteins showed greater divergence in the nucleotide sequence of VP1b than of VP1a, and the average divergences were 3.05% and 1.85%, respectively (Wilcoxon–Mann–Whitney test *p* < 0.05). However, greater divergence in amino acid sequences was observed in VP1a than in VP1b, and the average divergences were 1.05% and 0.60%, respectively (Wilcoxon–Mann–Whitney test *p* < 0.05) (Figure 4).

### 3.3. Phenuiviruses

All ticks collected in 2014 and 2015 were tested for phenuiviruses using pan-phlebovirus primers. The viral RNA of phenuiviruses was detected in three pools of *I. persulcatus* ticks collected in the forest area and in four pools of *D. reticulatus* ticks collected in the steppe area (Figure 1B, Table 1). The virus prevalence ranged from 0.66% to 6.95%.

For the phylogenetic analysis of phenuiviruses, various sequences were used: the viruses Gomselga and Stavropol detected in Chelyabinsk and other regions of Russia, the most related viruses detected around the world, and pathogenic tick-borne and sandfly/mosquito-borne phenuiviruses.

**Figure 6:**
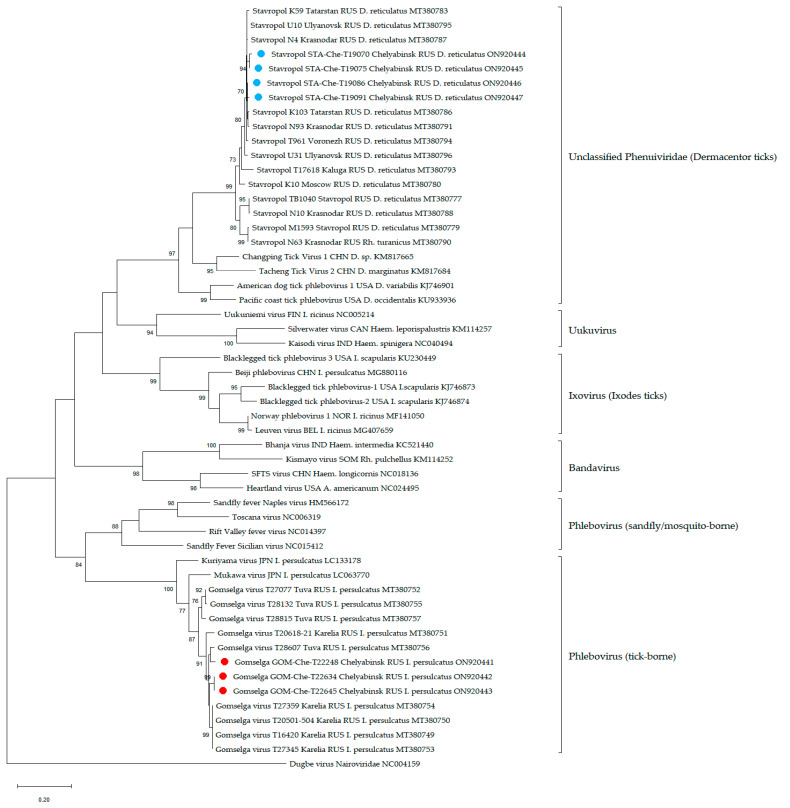
Phylogenetic analysis of the family Phenuiviridae. Phylogenetic trees were constructed using fragments of the L segment (474 bp) in MEGA X with the maximum likelihood method (1000 bootstrap replications). Bootstrap values (>70%) are shown at the branches. Blue circles—amplicons of the unclassified phenuivirus from the Chelyabinsk region described in this study. Red circles —amplicons of the phlebovirus from the Chelyabinsk region described in this study.

According to phylogenetic analysis, four amplicons from D. reticulatus belong to the Stavropol virus member of the unclassified Phenuiviridae (Figure 6, blue circle) and three amplicons from I. persulcatus belong to the Gomselga virus in the genus Phlebovirus (Figure 6, red circle).

## 4. Discussion

The main widespread arbovirus in Russia is TBEV, which is the cause of severe human disease [11]. Recently, an expansion of the TBEV area to the north has been noted [60]. The virus was detected in countries that were not endemic for TBE at the beginning of the 21st century, such as Norway [61,62], the United Kingdom [63,64,65], and the Netherlands [66,67]. In some endemic countries, new TBEV foci are emerging [68]. However, the expansion of the boundaries of the TBEV area occurs not only in the north but also to the south, wherein the emergence of TBEV in North Africa [69,70] and TBEV circulation in the steppe regions of Russia [51] have been noted.

The entire territory of the Chelyabinsk region is endemic for TBE [11,44]. The incidence is registered in the forest zone, forest-steppe, and steppe zones [43,44,46], while TBEV is detected only in forest and forest-steppe areas [47]. We collected and studied 3960 ticks (I. persulcatus—1275, Dermacentor ticks—2685). We isolated three TBEV strains from I. persulcatus ticks collected in the forest zone. All TBEV strains belong to the Zausaev group of the Siberian subtype. It was previously shown that it is mainly representatives of the Zausaev group of the Siberian subtype that circulate in the territory of the Chelyabinsk region [47,71], and all entries from Chelyabinsk region in GenBank belong to the Zausaev group of the Siberian subtype. Isolation of the Vasilchenko group TBEV in the Chelyabinsk region was also described, but there is no entry of this strain in GenBank [71]. We did not detect TBEV RNA in ticks of the genus Dermacentor. This is interesting because it was previously shown that in Russia and Europe, the infection rate of Dermacentor ticks was higher, or at least not lower, than that of Ixodes ticks [51,72,73], and TBEV titre was higher in Dermacentor ticks than in Ixodes ticks in experiments [74,75]. In the steppe zone of the Chelyabinsk region, there are forest belts along the roads, which I. persulcatus ticks inhabit. We believe that these ticks might be the cause of TBE cases in the steppe zone. However, we do not exclude the possibility that ticks of the genus Dermacentor might play some role in TBE morbidity in the steppe region, since it was previously shown that people suffered TBE after a bite by Dermacentor ticks [76]. We have previously shown that TBEV and YGTV can circulate simultaneously in the steppe regions of the Republic of Tuva [23,51]. We did not find TBEV in any place in the steppe zones of the Chelyabinsk region where YGTV occurred. Perhaps this is due to the fact that in the steppe regions of the Republic of Tuva the most common species is the D. nuttalli tick, in contrast to the Chelyabinsk region, where D. reticulatus and D. marginatus ticks inhabit the steppe regions.

Segmented flavi-like viruses were included in the group of unclassified viruses, related to the genus *Flavivirus* [9]. One of such viruses—ALSV—was first detected in China in a patient after a tick bite [7]. Moreover, ALSV was detected in mosquitos from China [7]; in ticks from Finland [25], France [24], and Russia [23,33]; and in mammals from China [30]. ALSV was detected in ticks collected in eight regions of Russia and, according to phylogenetic analysis of ALSV proteins VP1a and VP1b coding in segment 2, strains were divided into the “*I. ricinus*” and “*I. persulcatus*” groups, and, furthermore, the “*I. persulcatus*” group was divided into European and Asian subgroups [23]. In the Chelyabinsk region, we previously detected 11 ALSV-positive pools of *I. persulcatus* ticks and isolated six strains [23,33]. In this study, we additionally isolated one new strain named Salma15-T22545 from *I. persulcatus*, which clusters with other strains of ALSV from the Chelyabinsk region isolated from *I. persulcatus* ticks.

YGTV was first detected in *D. nuttalli* in the Xinjiang Uygur Autonomous Region of China, and sequences were deposited in GenBank in 2018. For the first time, we isolated the two strains of YGTV using tick cell lines of *I. ricinus* (IRE/CTVM19) and *H. anatolicum* (HAE/CTVM8) [23]. In the Chelyabinsk region we detected and isolated in the HAE/CTVM8 tick cell line a total of 26 strains of YGTV. Twenty-five strains were isolated from *Dermacentor* ticks collected in the steppe zone, and only one from *I. persulcatus* ticks collected in the forest zone. The highest infection rate with YGTV was noted in *D. reticulatus* ticks. Since *D. reticulatus* and *D. marginatus* ticks can be found not only in Russia but also in Asia, Europe, and North Africa [42,77], we can expect the circulation of this virus in other countries.

All of the above, as well as the fact that the previously described YGTV strains were detected mainly in ticks of the genus *Dermacentor*, may indicate that *Dermacentor* ticks can be considered the main vector of YGTV; as was the case for the Omsk haemorrhagic fever virus [10]. This assumption requires further research because it was shown that JMTV, under laboratory conditions, was not transmitted trans-stadially and was not found in the midgut and salivary glands of *Dermacentor silvarum* ticks [78]. According to the phylogenetic analysis, all strains from the Chelyabinsk region clustered together with the strains from China and the Republic of Altai. We did not find any relationship in the phylogenetic division of the YGTV strains, as was found for the geographic isolation of JMTV [79] and for the host species of ALSV [23]. This may have been due to the insufficient number of strains isolated in different territories.

It was previously shown that the NS5-like protein of ALSV encoding in segment 1 is the most divergent, and it is sometimes integrated in the *I. ricinus* tick genome [80]. The highest similarity was shown for a glycoprotein-coding segment 2 of ALSV [33]; therefore, in our work we used the sequences of segment 2 proteins VP1a and VP1b. It should be noted that there are more nucleotide substitutions in the VP1b protein than in the VP1a protein, while the number of non-synonymous nucleotide substitutions is higher in the VP1a protein for both ALSV and YGTV. The nucleotide sequence of the VP1a coding sequence contains two regions of high conservation [23] in places where it is overlapping with putative nuORF and VP1b frameshift site. Thus, high conservation of the nucleotide sequence is expected. At the same time, VP1a showed a higher rate of the non-synonymous nucleotide substitutions, which may indicate the potential importance of this region for the virus adaptation. Further study of the functions of these proteins and their structure will answer the question of why they differ so much.

We noticed that the nucleotide and amino acid sequences of VP1a and VP1b proteins of ALSV were more divergent than the same sequences of YGTV (Figure 4). The lower YGTV diversity compared to ALSV may be caused by the virus sampling bias because most of the YGTV strains in the analysed group were collected in the Chelyabinsk region. However, we cannot exclude the possibility of this being determined by the different ecologies of *Dermacentor* and *Ixodes* ticks [81,82,83,84].

In our work, we detected flavi-like viruses using primers for the genus *Flavivirus* and specific primers. In our previous work, we also detected both YGTV (in the Republic of Tuva) and ALSV (in the Republics of Tuva and Karelia) using pan-flavi primers [23,33]. However, as we see from our work and previous works [23,38], the number of flavi-like viruses detected using pan-flavi primers was lower than using specific ones. Pan-flavi primers were designed to detect representatives of the genus *Flavivirus* [52], at the time, when segmented flavi-like viruses were not known to exist. Although this system is able to occasionally detect flavi-like viruses, primers do not have enough complementary regions in segment 1 (NS5-like protein) of flavi-like viruses to reliably amplify their cDNA. Thus, virus-specific primers must be used for the reliable detection of the segmented flavi-like viruses and developing pan-flavi-like PCR assay may be necessary for quick and cheap analysis.

We found YGTV (strain Gubenka15-T22237 from *I. persulcatus*) and TBEV (strain Zlatoust15-T22241 from *I. persulcatus*) in one collection site. This fact, as well as the fact that the previously described ALSV and TBEV can be found in one location [38], may indicate that representatives of the genus *Flavivirus* and unclassified viruses, related to the genus *Flavivirus* may form combined foci.

According to a recently renewed classification, the *Phenuiviridae* family includes 20 genera, and viruses from 14 of them were detected in arthropods [35]. However, few viruses are associated with human diseases: sandfly/mosquito-borne phleboviruses (e.g., Naples phlebovirus, Rift Valley fever phlebovirus, Sicilian phlebovirus, and Toscana phlebovirus) and some tick-borne bandaviruses (Dabie bandavirus, Heartland bandavirus, and Bhanja bandavirus). Viruses from the remaining 12 genera of phenuiviruses should also be considered either as viruses of arthropods or as viruses with unknown epidemiologic potential.

The *Phlebovirus* genus mainly includes sandfly/mosquito-borne viruses; however, a small group of tick-borne viruses, such as Mukawa phlebovirus and Kuriyama phlebovirus, were also classified into this genus. The Gomselga virus detected in *I. persulcatus* ticks cluster with the tick-borne phleboviruses (Figure 6). Although the Mukawa phlebovirus and Kuriyama phlebovirus are phylogenetically close to the pathogenic sandfly/mosquito-borne viruses, there is only some serological evidence indicating the infection of wild animals with Mukawa phlebovirus, but no infectious virus was recovered upon the experimental infection [85]. The unclassified phenuivirus Stavropol is abundant on the territory of the European part of Russia and is highly specific to *D. reticulatus* ticks [37]. There are no data on the pathogenic properties of the Stavropol virus. Due to the unknown pathogenic potential of the viruses Gomselga and Stavropol, further studies of their distribution, properties, and interactions with other pathogens are needed.

The results of the current study are highly consistent with our previous publications regarding the widespread distribution of phenuiviruses and their high tick specificity across the European part of Russia [37,38]. In addition to the Chelyabinsk region, the Gomselga virus was recorded in the Republics of Karelia and Tuva for only *I. persulcatus* ticks, and its prevalence levels were 0.66–1.56%, 1.8–4.5%, and 2.0%, respectively. Conversely, Stavropol virus was detected in the Kaluga, Krasnodar, Moscow, Stavropol, Ulyanovsk, and Voronezh regions and in the Republic of Tatarstan [37], almost exclusively in *D. reticulatus* ticks. A previously reported prevalence of the Stavropol virus in the Chelyabinsk region (6.95%) was lower than in other regions of Russia [37], especially in the Ulyanovsk (21.5%) and Tatarstan (31.0%) regions. On the other hand, a relatively high prevalence and high specificity of YGTV to *D. reticulatus* ticks may imply that there is competition or at least interaction between YGTV and the Stavropol virus. Additional data regarding the prevalence of YGTV in other regions, compared with prevalence of the Stavropol virus, would allow us to speculate about this fact.

## 5. Conclusions

We showed in this study that (1) TBEV is spread in the forest zone of the Chelyabinsk region; (2) YGTV is widespread in the steppe and forest-steppe zones and is mainly associated with ticks of the genus *Dermacentor*; (3) there are combined foci of TBEV and YGTV on the territory of the Chelyabinsk region; (4) ALSV is spread in the forest zone and is associated with *I. persulcatus* ticks; (5) the phlebovirus Gomselga and unclassified phenuivirus Stavropol are spread in the Chelyabinsk region and associated with *I. persulcatus* and *D. reticulatus* ticks, respectively. The virus prevalence of the unclassified phenuivirus Stavropol in the Chelyabinsk region is lower than in neighbouring regions.

## Figures and Tables

**Table 1:** Prevalence of the TBEV, flavi-like, and phenuiviruses in ticks in the Chelyabinsk region in the years 2014–2015 ^a^.

Latitude, Longitude	Tick Species	Number of Analysed Ticks (Total)	Number of Analysed Pools	Number of Virus-Positive Pools	Prevalence ^a^, % (CI 95%)	Virus, Strains
53.881103, 59.163164	*D. reticulatus*	65	15	4	6.95 (2.21–15.50)	Stavropol, STA-Che-T19070 ^b^
						Stavropol, STA-Che-T19075 ^b^
						Stavropol, STA-Che-T19086 ^b^
						Stavropol, STA-Che-T19091 ^b^
	*D. marginatus*	3	1	0	0	-
53.382567, 59.9295	*D. reticulatus*	11	4	2	21.74 (3.93–54.60) ^c^	YGTV, Fershampenuaz14-T19014
						YGTV, Paris14-T19197
	*D. marginatus*	39	9	0	0	-
	*I. persulcatus*	1	1	0	0	-
52.885633, 60.051833	*D. reticulatus*	125	26	3	2.52 (0.63–6.42)	YGTV, Kartaly14-T19309
						YGTV, Kartaly14-T19314
						YGTV, Kartaly14-T19551
	*D. marginatus*	112	23	2	1.85 (0.31–5.61)	YGTV, Kartaly14-T19623
						YGTV, Kartaly14-T19658
	*I. persulcatus*	2	1	0	0	-
52.940933, 59.935533	*D. reticulatus*	7	2	1	17.02 (1.05–57.59) ^c^	YGTV, Kartaly14-T19346
52.053183, 59.957667	*D. reticulatus*	6	2	0	0	-
	*D. marginatus*	130	27	1	0.78 (0.04–3.39)	YGTV, Bredy14-T19463
52.497667, 60.00035	*D. reticulatus*	21	5	2	11.70 (2.02–32.62) ^c^	YGTV, Bredy14-T19736
						YGTV, Bredy14-T19741
	*D. marginatus*	30	6	0	0	-
52.47595, 59.871383	*D. reticulatus*	14	3	2	20.47 (3.60–54.09) ^c^	YGTV, Bredy14-T19763
						YGTV, Bredy14-T19767
	*D. marginatus*	6	2	0	0	-
52.459233, 60.249483	*D. reticulatus*	3	1	0	0	-
	*D. marginatus*	53	11	1	1.96 (0.11–8.36)	YGTV, Bredy14-T19813
55.02145, 60.168283	*I. persulcatus*	169	33	9	6.00 (2.93–10.59)	**ALSV, Miass501 ^b^**
						**ALSV, Miass502**
						**ALSV, Miass506**
						**ALSV, Miass508 ^b^**
						**ALSV, Miass510 ^b^**
						**ALSV, Miass515 ^b^**
						**ALSV, Miass519**
						**ALSV, Miass523 ^b^**
						**ALSV, Miass527**
52.468017, 60.226217	*D. reticulatus*	102	26	4	4.18 (1.31–9.45)	YGTV, Bredy15-T22173
						YGTV, Bredy15-T22181
						YGTV, Bredy15-T22188
						YGTV, Bredy15-T22189
	*D. marginatus*	24	7	0	0	-
52.494667, 60.011817	*D. reticulatus*	14	4	0	0	-
	*D. marginatus*	27	7	1	3.93 (0.23–16.22)	YGTV, Bredy15-T22208
52.970983, 60.604833	*D. reticulatus*	15	4	1	7.17 (0.42–28.07) ^c^	YGTV, Kartaly15-T22141
	*D. marginatus*	16	4	0	0	-
54.39845, 60.783167	*D. reticulatus*	130	34	2	1.58 (0.26–4.78)	YGTV, Plast15-T22436
						YGTV, Plast15-T22438
	*D. marginatus*	15	4	1	7.17 (0.42–28.07) ^c^	YGTV, Plast15-T22415
54.569117, 60.288267	*D. reticulatus*	24	6	0	0	-
	*D. marginatus*	118	30	1	0.86 (0.05–3.72)	YGTV, Mir15-T22470
54.527867, 60.334233	*D. reticulatus*	5	2	0	0	-
	*D. marginatus*	61	16	1	1.68 (0.10–7.19)	YGTV, Zaozernyy15-T22264
	*I. persulcatus*	4	2	0		-
55.209033, 59.571467	*I. persulcatus*	133	26	1	0.76 (0.04–3.32)	TBEV, Zlatoust15-T22637
				2	1.56 (0.26–4.73)	Gomselga, GOM-Che-T22634 ^b^
						Gomselga, GOM-Che-T22645 ^b^
55.22005, 59.56005	*I. persulcatus*	154	29	1	0.66 (0.04–2.87)	YGTV, Gubenka15-T22237
				1	0.66 (0.04–2.87)	TBEV, Zlatoust15-T22241
				1	0.66 (0.04–2.87)	Gomselga, GOM-Che-T22248 ^b^
55.021583, 60.169783	*I. persulcatus*	76	14	2	2.78 (0.47–8.35)	**ALSV, Miass15-T22516**
						**ALSV, Miass15-T22517**
55.357683, 59.51585	*I. persulcatus*	46	9	1	2.28 (0.13–9.64)	TBEV, Kusa15-T22532
56.168967, 60.471233	*I. persulcatus*	90	18	1	1.13 (0.06–4.88)	ALSV, Salma15-T22545

Strains/amplicons that have been described previously [23,33] are highlighted in bold font. ^a^ The prevalence was estimated for variable pool sizes with the assumption of 100% test sensitivity and specificity. ^b^ Amplicon is the primary material containing viral RNA fragments with the identified nucleotide sequences without strain isolation. ^c^ Hypothetical data, due to the small number of analysed ticks.

## Data Availability

The data presented in this study are available in the article and supplementary material. Obtained sequencing data were deposited in the GenBank database: tick-borne encephalitis virus (OM056525–OM056527), Yanggou tick virus (OP125777–OP125799, ON448356, ON448367, ON448374), Alongshan virus (OP125776), Gomselga virus (ON920441–ON920443), and Stavropol virus (ON920444–ON920447).

## Supplementary Materials

The following supporting information can be downloaded at: https://www.mdpi.com/article/10.3390/v14122699/s1, Table S1: Locations of tick collection; Table S2: Specific primers for amplification and sequencing of the segment 2 of the Yanggou tick virus genome; Table S3: Percent identity of the complete nucleotide and amino acid sequences of protein E of the three TBEV strains; Table S4: Percent identity of the nucleotide sequences of the protein VP1a of 29 strains of the Yanggou tick virus; Table S5: Percent identity of the amino acid sequences of the protein VP1a of 29 strains of the Yanggou tick virus; Table S6: Percent identity of the nucleotide sequences of the protein VP1b of 16 strains of the Yanggou tick virus; Table S7: Percent identity of the amino acid sequences of the protein VP1b of 16 strains of the Yanggou tick virus; Table S8: Percent identity of the nucleotide sequences of the protein VP1a of 16 strains of the Alongshan virus; Table S9: Percent identity of the amino acid sequences of the protein VP1a of 16 strains of the Alongshan virus; Table S10: Percent identity of the nucleotide sequences of the protein VP1b of 16 strains of the Alongshan virus; Table S11: Percent identity of the amino acid sequences of the protein VP1b of 16 strains of the Alongshan virus; Figure S1: Phylogenetic analysis of the complete nucleotide sequence of the Yanggou tick virus VP1a protein; Figure S2: Phylogenetic analysis of the complete amino acid sequence of the Yanggou tick virus VP1a protein; Figure S3: Phylogenetic analysis of the complete nucleotide sequence of the Yanggou tick virus VP1b protein; Figure S4: Phylogenetic analysis of the complete amino acid sequence of the Yanggou tick virus VP1b protein.
