# Supplementary material for: Distribution and Characterisation of Tick-Borne Flavi-, Flavi-like, and Phenuiviruses in the Chelyabinsk Region of Russia
Source: Viruses. 2022 Dec 1;14(12):2699. doi: 10.3390/v14122699 (PMC9780909; doi:10.3390/v14122699)
Supplement: Supplementary file 1 [file viruses-14-02699-s001.zip › viruses-2015890-supplementary.pdf]

Table S1. Locations of tick collection

Latitude	Longitude	Date (mm.year)	Tick species				Collection method
			I. persulcatus (♂+♀)	*I. persulcatus.* NN	*D. reticulatus* (♂+♀)	*D. marginatus* (♂+♀)	
54.083520	59.546226	05.2014	2+1		9+8	2+10	FV
54.077678	59.557866	05.2014			0+3	0+2	FV
54.055812	59.605618	05.2014	1+7		0+2	5+12	FV
54.048115	59.662730	05.2014				9+15	FV
54.133282	59.531905	05.2014	1+0			1+7	FV
54.116667	59.533333	05.2014	1+0				FV
54.124452	59.546685	05.2014	29+30		0+5	6+3	FV
53.874638	59.229960	05.2014			5+4	30+30	RA
53.855183	59.240433	05.2014			22+36	33+55	FV
53.880754	59.241434	05.2014				24+48	RA
53.857781	59.242444	05.2014			0+3	2+2	FV
53.881783	59.1628	05.2014			50+106	18+33	FV
53.881103	59.163164	05.2014			21+45	1+2	FV
54.131565	59.541217	05.2014	15+8			0+1	FV
53.674117	59.731317	05.2014			3+1	21+25	FV
53.599617	59.82275	05.2014				0+2	FV
53.524360	59.799737	05.2014				9+27	RA
53.5159	59.775067	05.2014			2+0	38+63	FV
53.382567	59.9295	05.2014	0+1		3+8	17+22	FV
53.3565	60.377167	05.2014				2+3	RA
52.885633	60.051833	05.2014	1+1		41+84	34+78	FV
52.940933	59.935533	05.2014			4+3		FV
52.053183	59.957667	05.2014			3+3	39+91	FV
53.124567	59.896933	05.2014			2+3	1+2	FV
53.14555	59.935283	05.2014	4+0		5+4	0+2	FV
53.315983	60.1329	05.2014			1+0		FV
53.233183	60.535117	05.2014	0+1		2+5	0+1	FV
52.82475	60.5678	05.2014				0+2	FV
52.428583	60.306967	05.2014				0+1	FV
52.49865	60.14015	05.2014				5+1	RA
52.497667	60.00035	05.2014			11+10	10+20	FV
52.47595	59.871383	05.2014			1+13	1+5	FV
52.459233	60.249483	05.2014			1+2	22+31	FV
52.541683	60.394067	05.2014			0+1		FV
54.446533	60.79435	05.2014				3+3	FV
54.629017	60.650567	05.2014	1+1			0+3	FV
55.128629	60.072396	05.2014	19+24				FV
55.02145	60.168283	05.2014	75+78	13			FV
54.410216	62.698490	05.2014			21+38	11+16	FV
53.24225	60.473233	05.2015			0+1	0+1	FV
53.266383	60.461617	05.2015			2+6	6+23	FV
53.214733	60.572467	05.2015			0+2	2+4	FV
52.945	60.86385	05.2015				3+16	FV
52.918083	60.847017	05.2015				4+6	FV
52.427433	60.307167	05.2015				0+1	FV
52.452917	60.26015	05.2015			1+2	2+8	FV
52.468017	60.226217	05.2015			34+68	9+15	FV

52.494667	60.011817	05.2015			4+10	8+19	FV
52.970983	60.604833	05.2015			3+12	8+8	FV
53.066141	60.660224	05.2015				0+1	FV
53.067168	60.669646	05.2015				7+8	RA
53.5737	60.764017	05.2015	1+1		2+21	4+7	FV
53.719583	60.727567	05.2015			6+48	60+111	FV
54.44595	60.70385	05.2015	8+6		0+3	0+2	FV
54.441733	60.71415	05.2015	0+3		6+13	6+6	FV
54.451733	60.751667	05.2015	1+1		6+14	5+4	FV
54.435233	60.788817	05.2015			0+2		FV
54.39845	60.783167	05.2015			41+89	4+11	FV
54.569117	60.288267	05.2015			7+15	43+75	FV
54.568483	60.284117	05.2015	0+1		12+17	7+11	FV
54.527867	60.334233	05.2015	1+3		2+3	15+46	FV
54.54835	59.947367	05.2015			7+10	29+49	FV
54.577033	59.966133	05.2015	4+3			1+5	FV
55.153347	59.698556	05.2015	0+1				FV
55.209033	59.571467	05.2015	71+62				FV
55.22005	59.56005	05.2015	77+77				FV
55.021583	60.169783	05.2015	30+42	5			FV
55.01885	60.165117	05.2015	6+5				FV
55.362883	59.556017	05.2015	6+3				FV
55.357683	59.51585	05.2015	21+25				FV
55.791433	60.617467	05.2015	7+4				FV
56.008567	60.524933	05.2015	5+10				FV
56.0172	60.5847	05.2015	30+26				FV
55.969983	60.632067	05.2015	12+9				FV
55.900983	60.7282	05.2015	2+1				FV
55.891833	60.693683	05.2015	26+18				FV
55.89465	60.695333	05.2015	29+29				FV
56.161217	60.474183	05.2015	17+24				FV
56.167933	60.4683	05.2015	25+33	1			FV
56.168967	60.471233	05.2015	40+47	3			FV
56.2076	60.443033	05.2015	46+53				FV
**Total:**			**614+639**	**22**	**335+719**	**490+953**	**FV**
					**5+4**	**77+102**	**RA**

Grey color – ticks described in previous article [1]

RA – removed from animal, **FV** – flagging vegetation

♂ - adult male, ♀ - adult female, NN - nymphs

Table S2. Specific primers for amplification and sequencing of the segment 2 of the Yanggou tick virus genome.

Name of the primer	Nucleotide sequence (5′ – 3′)	Primer direction	Genome locus	Amplicon size, bp	Temperature, ⁰C	Reference
**Yanggou tick virus**						
Yanggou_gly_1F	ACTACTGGTTGCCGTCCTCG	forward	segment 2	305	52	this study
Yanggou_gly_1R	GTCGCTGCAGTCAAATATCT	reverse				
Yanggou_seg2_1F	GACAGAATCCAAAGACGACAC	forward	segment 2	791	50	
Yanggou_seg2_1R	CACACTGTCCCGTTCATCCA	reverse				
Yanggou_seg2_2F	TGACAAACCAGATGCGGGAA	forward	segment 2	1184	52	
Yanggou_seg2_2R	GCAGCAAGTTGGAGAATGCC	reverse				
Yanggou_seg2_4F	GGATGGGGTGGTCAGAGTC	forward	segment 2	869	52	
Yanggou_seg2_4R	GTTAATCCGGGCGTCATCT	reverse				
**Alongshan virus**						
Miass_gly_3F	TGGATCAGCTCACACCACAC	forward	segment 2	333	53	[2]
Miass_gly_3R	TCACCGTCACAGTGGAATGG	reverse				
**Tick-borne encephalitis virus**						
Kgg31	AAAGGCAGCATTGTGACCTG	forward	E protein	361	52	[3]
Kgg19	CGTGTCTCCACGGCAGAGCC	reverse				
Kgg65	AGATTTTCTTGCACGT	forward	5′ NTR	1100	50	[4]
TBE1095r	G(C/a)GTCAAGCCACACATCC	reverse	E protein			
Kgg35	CTGACCGTGGAAAGTGTGG	forward	M protein	1500	55	
Kgg26	AAGCTCATGGACATGGTAGG	reverse	E protein			
Kgg16	AGGGGAGCAGCATTGGAAG	forward	E protein	1200	55	
Kgg30	TGGTGCTCCTCACAGAAGC	reverse	NS1 protein			
MAMD	AACATGATGGGRAARAGRGA RAA	forward	NS5 protein	250	50	[5]
cFD2	GTGTCCCAGCCGGCGGTGTC ATCAGC	reverse				
**Phenuiviruses**						
PhlP2	GGCTACTTCAARAAYAARGA NGA	forward	L segment	507	50	[6]
PhlM2	CTCTCTCAGICCICCRTGYTG	reverse				

Table S3. Percent identity of the complete nucleotide and amino acid sequences of protein E of the three TBEV strains. Genbank accession numbers for each strain are as follows: strain Kusa15-T22532 (OM056525), strain Zlatoust15-T22637 (OM056526), strain Zlatoust15-T22241 (OM056527).

	Nucleotide identity, %		Amino acid identity, %	
Strain	Zlatoust15-T22637	Zlatoust15-T22241	Zlatoust15-T22637	Zlatoust15-T22241
Kusa15-T22532	99.12	96.31	99.6	99.4
Zlatoust15-T22637		96.81		99.4

Table S4. Percent identity of the nucleotide sequences of the protein VP1a of 16 strains of the Alongshan virus. GenBank accession numbers for each strain are presented in the table.

Strain_GenBank accession number	Kuutsalo-23_MN107154	Haapasaari-18_MN107158	JMTV/I.ricinus/France_ MN095520	Rowan19-T32778_MW525310	Kursh18-T30290_MW525309	Kursh18-T30284_MW525306	Ulya15-T22688_MW525312	Tat14-T21924_MW525313	Goms13-T17158_MW525287	Galozero14-T20426_MN604229	Miass519_MN648776	Miass506_MW525319	H3_MH158416	Erjey17-T25134_MW525295	Miass527_MN648772
Haapasaari-18_MN107158	98.53														
JMTV/I.ricinus/France_MN095520	98.67	99.02													
Rowan19-T32778_MW525310	98.88	98.95	99.09												
Kursh18-T30290_MW525309	99.09	99.16	99.30	99.65											
Kursh18-T30284_MW525306	97.38	97.60	98.03	97.96	97.89										
Ulya15-T22688_MW525312	95.76	95.69	95.99	95.91	95.99	95.91									
Tat14-T21924_MW525313	95.98	95.91	96.06	96.13	96.21	95.99	99.09								
Goms13-T17158_MW525287	92.99	92.99	93.69	93.31	93.07	93.77	93.24	92.69							
Galozero14-T20426_MN604229	93.61	93.84	94.16	93.93	93.69	93.93	93.78	93.70	95.69						
Miass519_MN648776	93.54	94.24	94.55	94.32	94.09	94.62	93.63	93.55	95.84	96.06					
Miass506_MW525319	93.54	94.24	94.55	94.32	94.09	94.62	93.63	93.55	95.84	96.06	100.00				
H3_MH158416	91.27	91.44	91.77	91.69	91.45	92.33	91.78	91.70	91.71	92.50	92.89	92.89			
Erjey17-T25134_MW525295	92.32	92.49	93.12	93.05	92.81	93.06	92.74	92.82	92.99	93.29	93.83	93.83	94.91		
Miass527_MN648772	92.48	92.65	93.28	93.36	93.13	92.91	92.90	92.66	92.98	92.98	93.37	93.37	94.76	98.74	
Miass502_MW525315	92.65	92.66	93.29	93.37	93.13	93.07	93.06	92.82	93.30	93.15	93.53	93.53	94.91	98.74	99.72

Analyses were conducted using the Maximum Composite Likelihood model [7]. This analysis involved 16 nucleotide sequences. Codon positions included were 1st+2nd+3rd+Noncoding. All positions containing gaps and missing data were eliminated (complete deletion option). There were a total of 1446 positions in the final dataset. Evolutionary analyses were conducted in MEGA X [8]. Colour indicates from what tick species the strain was isolated: green – *Ixodes ricinus*, red – *Ixodes persulcatus*.

Table S5. Percent identity of the amino acid sequences of the protein VP1a of 16 strains of the Alongshan virus. GenBank accession numbers for each strain are presented in the table.

Strain_GenBank accession number	Kuutsalo-23_MN107154	Haapasaari-18_MN107158	JMTV/I.ricinus/France_MN095520	Rowan19-T32778_MW525310	Kursh18-T30290_MW525309	Kursh18-T30284_MW525306	Ulya15-T22688_MW525312	Tat14-T21924_MW525313	Goms13-T17158_MW525287	Galozero14-T20426_MN604229	Miass519_MN648776	Miass506_MW525319	H3_MH158416	Erjey17-T25134_MW525295	Miass527_MN648772
Haapasaari-18_MN107158	99.16														
JMTV/I.ricinus/France_MN095520	99.37	99.37													
Rowan19-T32778_MW525310	98.96	98.96	99.58												
Kursh18-T30290_MW525309	99.37	99.37	100.00	99.58											
Kursh18-T30284_MW525306	97.90	97.90	98.53	98.53	98.53										
Ulya15-T22688_MW525312	96.19	96.62	96.40	96.40	96.40	96.62									
Tat14-T21924_MW525313	96.19	96.62	96.40	96.40	96.40	96.62	99.58								
Goms13-T17158_MW525287	94.88	94.88	95.54	95.54	95.54	95.75	95.32	95.32							
Galozero14-T20426_MN604229	94.88	94.88	95.10	95.54	95.10	94.88	95.32	95.32	97.47						
Miass519_MN648776	95.32	95.32	95.97	95.97	95.97	95.75	95.32	95.32	97.90	97.47					
Miass506_MW525319	95.32	95.32	95.97	95.97	95.97	95.75	95.32	95.32	97.90	97.47	100.00				
H3_MH158416	93.78	93.78	94.00	94.00	94.00	94.66	95.10	95.10	96.83	96.40	96.40	96.40			
Erjey17-T25134_MW525295	93.78	93.78	94.44	94.44	94.44	94.22	94.66	94.66	97.26	96.62	96.83	96.83	97.90		
Miass527_MN648772	93.78	93.78	94.44	94.44	94.44	93.78	93.78	93.78	96.83	95.75	95.97	95.97	97.26	99.16	
Miass502_MW525315	94.22	94.22	94.88	94.88	94.88	94.22	94.22	94.22	97.26	96.19	96.40	96.40	97.69	99.16	99.58

Analyses were conducted using the Poisson correction model [9]. This analysis involved 16 amino acid sequences. The coding data was translated assuming a Standard genetic code table. All positions containing gaps and missing data were eliminated (complete deletion option). There were a total of 481 positions in the final dataset. Evolutionary analyses were conducted in MEGA X [8]. Colour indicates from what tick species the strain was isolated: green – *Ixodes ricinus*, red – *Ixodes persulcatus*.

Table S6. Percent identity of the nucleotide sequences of the protein VP1b of 16 strains of the Alongshan virus. GenBank accession numbers for each strain are presented in the table.

Strain_GenBank accession number	Kuutsalo-23_MN107154	Haapasaari-18_MN107158	JMTV/I.ricinus/France_MN095520	Rowan19-T32778_MW525310	Kursh18-T30290_MW525309	Kursh18-T30284_MW525306	Ulya15-T22688_MW525312	Tat14-T21924_MW525313	Goms13-T17158_MW525287	Galozero14-T20426_MN604229	Miass519_MN648776	Miass506_MW525319	H3_MH158416	Erjey17-T25134_MW525295	Miass527_MN648772
Haapasaari-18_MN107158	98.76														
JMTV/I.ricinus/France_MN095520	98.55	98.34													
Rowan19-T32778_MW525310	99.07	98.66	98.66												
Kursh18-T30290_MW525309	98.87	98.24	98.24	98.77											
Kursh18-T30284_MW525306	96.52	96.41	96.41	96.74	96.63										
Ulya15-T22688_MW525312	93.70	93.83	93.83	93.48	94.05	93.60									
Tat14-T21924_MW525313	93.81	94.05	94.05	93.70	94.05	93.25	98.97								
Goms13-T17158_MW525287	92.79	92.56	93.25	92.68	93.03	91.98	91.37	91.47							
Galozero14-T20426_MN604229	92.22	92.33	92.21	92.11	92.23	91.85	91.22	91.21	94.53						
Miass519_MN648776	93.49	93.49	93.72	93.38	93.95	93.61	91.84	91.59	95.10	95.31					
Miass506_MW525319	93.49	93.49	93.72	93.38	93.95	93.61	91.84	91.59	95.10	95.31	100.00				
H3_MH158416	89.86	89.87	89.62	90.01	90.60	89.53	89.22	89.57	90.40	89.01	89.65	89.65			
Erjey17-T25134_MW525295	90.36	89.89	90.61	90.98	90.86	90.26	89.48	90.07	90.76	89.90	90.87	90.87	92.93		
Miass527_MN648772	90.58	90.11	90.83	90.71	91.31	89.99	89.33	89.93	90.98	89.63	90.38	90.38	92.68	98.13	
Miass502_MW525315	90.04	89.56	90.29	90.17	90.54	89.44	88.52	89.12	90.20	89.32	89.95	89.95	91.81	97.38	98.86

(green)	*Ixodes ricinus*	(red)	*Ixodes persulcatus*

Analyses were conducted using the Maximum Composite Likelihood model [7]. This analysis involved 16 nucleotide sequences. Codon positions included were 1st+2nd+3rd+Noncoding. All positions containing gaps and missing data were eliminated (complete deletion option). There were a total of 981 positions in the final dataset. Evolutionary analyses were conducted in MEGA X [8].

Table S7. Percent identity of the amino acid sequences of the protein VP1b of 16 strains of the Alongshan virus. GenBank accession numbers for each strain are presented in the table.

Strain_GenBank accession number	Kuutsalo-23_MN107154	Haapasaari-18_MN107158	JMTV/I.ricinus/France_ MN095520	Rowan19-T32778_MW525310	Kursh18-T30290_MW525309	Kursh18-T30284_MW525306	Ulya15-T22688_MW525312	Tat14-T21924_MW525313	Goms13-T17158_MW525287	Galozero14-T20426_MN604229	Miass519_MN648776	Miass506_MW525319	H3_MH158416	Erjey17-T25134_MW525295	Miass527_MN648772
Haapasaari-18_MN107158	100.00														
JMTV/I.ricinus/France_MN095520	100.00	100.00													
Rowan19-T32778_MW525310	100.00	100.00	100.00												
Kursh18-T30290_MW525309	100.00	100.00	100.00	100.00											
Kursh18-T30284_MW525306	99.69	99.69	99.69	99.69	99.69										
Ulya15-T22688_MW525312	99.38	99.38	99.38	99.38	99.38	99.08									
Tat14-T21924_MW525313	99.69	99.69	99.69	99.69	99.69	99.38	99.69								
Goms13-T17158_MW525287	98.45	98.45	98.45	98.45	98.45	98.14	98.14	98.14							
Galozero14-T20426_MN604229	99.08	99.08	99.08	99.08	99.08	98.77	98.45	98.77	98.14						
Miass519_MN648776	99.38	99.38	99.38	99.38	99.38	99.08	98.77	99.08	98.45	99.08					
Miass506_MW525319	99.38	99.38	99.38	99.38	99.38	99.08	98.77	99.08	98.45	99.08	100.00				
H3_MH158416	97.83	97.83	97.83	97.83	97.83	97.52	97.20	97.52	97.52	97.52	97.83	97.83			
Erjey17-T25134_MW525295	97.52	97.52	97.52	97.52	97.52	97.20	96.88	97.20	97.20	97.20	97.52	97.52	99.08		
Miass527_MN648772	97.20	97.20	97.20	97.20	97.20	96.88	96.57	96.88	96.88	96.88	97.20	97.20	98.77	99.08	
Miass502_MW525315	96.57	96.57	96.57	96.57	96.57	96.25	95.93	96.25	96.25	96.25	96.57	96.57	98.14	98.45	98.77

(green)	*Ixodes ricinus*	(red)	*Ixodes persulcatus*

Analyses were conducted using the Poisson correction model [9]. This analysis involved 16 amino acid sequences. The coding data was translated assuming a Standard genetic code table. All positions containing gaps and missing data were eliminated (complete deletion option). There were a total of 326 positions in the final dataset. Evolutionary analyses were conducted in MEGA X [8].

Table S8. Percent identity of the nucleotide sequences of the protein VP1a of 29 strains of the Yanggou tick virus. GenBank accession numbers for each strain are presented in the table.

Strain_GenBank accession number	16-T2_ MH688533	17-L1_ MH688537	Erzin14-T20074_ MW525323	XJ-YGTV-1_ MT248419	YG_ MH688530	Republic_Altay/997/2016_ MW556731	Republic_Altay/1001/2016_ MW556735	Bredy14-T19813_ OP125785	Bredy14-T19463_OP125781	Mir15-T22470_ OP125797	Zaozernyy15-T22264_ OP125799	Bredy15-T22208_ ON448367	Plast15-T22415_ ON448374	Bredy14-T19767_ OP125784	Bredy14-T19736_ OP125782	Bredy14-T19741_ OP125783	Kartaly14-T19346_ OP125793	Kartaly14-T19309_ OP125791	Kartaly14-T19314_ OP125792	Kartaly14-T19658_ OP125795	Fershampenuaz14- T19014_OP125789	Kartaly14-T19551_ OP125794	Bredy15-T22181_ OP125786	Bredy15-T22188_ OP125787	Bredy15-T22189_ OP125788	Kartaly15-T22141_ OP125796	Plast15-T22438_ OP125798	Plast15-T22436_ ON448356
17-L1_MH688537	99.62																											
Erzin14-T20074_MW525323	94.51	94.77																										
XJ-YGTV-1_MT248419	96.25	96.66	95.68																									
YG_MH688530	99.08	99.01	94.93	97.06																								
Republic_Altay/997/2016_MW556731	98.00	98.08	95.10	96.41	98.39																							
Republic_Altay/1001/2016_MW556735	98.00	98.08	95.10	96.41	98.39	100.00																						
Bredy14-T19813_OP125785	97.92	98.00	94.93	95.77	98.16	98.39	98.39																					
Bredy14-T19463_OP125781	97.13	97.37	95.68	96.10	97.53	97.92	97.92	97.53																				
Mir15-T22470_OP125797	96.65	96.89	95.43	95.86	97.05	97.45	97.45	97.21	98.62																			
Zaozernyy15-T22264_OP125799	97.13	97.37	95.76	96.18	97.52	97.92	97.92	97.68	99.08	99.54																		
Bredy15-T22208_ON448367	97.21	97.45	95.84	96.26	97.60	97.84	97.84	97.45	99.01	99.32	99.77																	
Plast15-T22415_ON448374	97.13	97.37	95.76	96.18	97.52	97.92	97.92	97.68	99.08	99.54	100.00	99.77																
Bredy14-T19767_OP125784	97.28	97.52	95.92	96.34	97.68	97.92	97.92	97.52	98.93	99.24	99.70	99.92	99.70															
Bredy14-T19736_OP125782	96.81	97.05	95.59	95.86	97.20	97.60	97.60	97.37	98.78	99.24	99.70	99.47	99.70	99.39														
Bredy14-T19741_OP125783	97.05	97.29	95.67	96.10	97.44	97.84	97.84	97.60	99.01	99.47	99.92	99.70	99.92	99.62	99.62													
Kartaly14-T19346_OP125793	96.41	96.65	95.19	95.77	96.97	97.21	97.21	97.13	98.39	98.78	99.24	99.01	99.24	98.93	99.09	99.16												
Kartaly14-T19309_OP125791	97.29	97.37	96.00	96.10	97.68	97.92	97.92	97.53	98.62	98.31	98.78	98.70	98.78	98.78	98.47	98.70	98.08											
Kartaly14-T19314_OP125792	97.45	97.69	96.00	96.42	97.84	98.23	98.23	97.84	99.70	98.62	99.08	99.01	99.08	99.08	98.78	99.01	98.39	98.93										
Kartaly14-T19658_OP125795	97.45	97.53	96.16	96.26	97.84	98.08	98.08	97.69	98.78	98.47	98.93	98.85	98.93	98.93	98.62	98.85	98.24	99.85	99.09									
Fershampenuaz14-T19014_OP125789	96.56	96.81	95.35	95.61	96.96	97.52	97.52	97.13	98.70	99.01	99.47	99.24	99.47	99.16	99.16	99.39	98.70	98.23	98.54	98.39								
Kartaly14-T19551_OP125794	96.97	97.21	95.59	96.02	97.36	97.76	97.76	97.53	98.93	99.39	99.85	99.62	99.85	99.54	99.54	99.92	99.09	98.62	98.93	98.78	99.32							
Bredy15-T22181_OP125786	97.05	97.29	95.84	96.26	97.44	97.84	97.84	97.45	98.85	99.16	99.62	99.54	99.62	99.62	99.32	99.54	98.86	98.70	99.01	98.85	99.09	99.47						
Bredy15-T22188_OP125787	97.05	97.29	95.67	96.10	97.44	97.84	97.84	97.76	99.01	99.47	99.92	99.70	99.92	99.62	99.62	99.85	99.16	98.70	99.01	98.85	99.39	99.77	99.54					
Bredy15-T22189_OP125788	96.73	96.97	95.51	95.93	97.12	97.68	97.68	97.29	98.85	99.01	99.47	99.24	99.47	99.16	99.16	99.39	98.70	98.39	98.70	98.54	99.54	99.32	99.09	99.39				
Kartaly15-T22141_OP125796	96.65	96.89	95.43	95.70	97.05	97.45	97.45	97.21	98.62	99.24	99.55	99.32	99.55	99.24	99.54	99.47	98.93	98.31	98.62	98.47	99.01	99.39	99.16	99.47	99.01			
Plast15-T22438_OP125798	96.49	96.73	95.27	95.69	96.89	97.29	97.29	97.05	98.47	99.85	99.39	99.16	99.39	99.09	99.09	99.32	98.63	98.31	98.47	98.47	98.86	99.24	99.01	99.32	98.86	99.09		
Plast15-T22436_ON448356	96.96	97.21	95.42	95.93	97.36	97.76	97.76	97.52	98.78	99.16	99.62	99.39	99.62	99.32	99.32	99.54	99.01	98.47	98.78	98.62	99.08	99.47	99.24	99.54	99.08	99.16	99.01	
Gubenka15-T22237_OP125790	97.13	97.37	95.76	96.18	97.52	97.92	97.92	97.68	99.08	99.54	100.00	99.77	100.00	99.70	99.70	99.92	99.24	98.78	99.08	98.93	99.47	99.85	99.62	99.92	99.47	99.55	99.39	99.62

Analyses were conducted using the Maximum Composite Likelihood model [7]. This analysis involved 29 nucleotide sequences. Codon positions included were 1st+2nd+3rd+Noncoding. All positions containing gaps and missing data were eliminated (complete deletion option). There were a total of 1323 positions in the final dataset.

Evolutionary analyses were conducted in MEGA X [8]. Colour indicates from what tick species the strain was isolated: green – *Dermacentor nuttalli*, blue – *Dermacentor marginatus*, orange – *Dermacentor reticulatus*, red – *Ixodes persulcatus*.

Table S9. Percent identity of the amino acid sequences of the protein VP1a of 29 strains of the Yanggou tick virus. GenBank accession numbers for each strain are presented in the table.

Strain_GenBank accession number	16-T2_ MH688533	17-L1_ MH688537	Erzin14-T20074_ MW525323	XJ-YGTV-1_ MT248419	YG_ MH688530	Republic_Altay/997/2016_ MW556731	Republic_Altay/1001/2016_ MW556735	Bredy14-T19813_ OP125785	Bredy14-T19463_OP125781	Mir15-T22470_ OP125797	Zaozernyy15-T22264_ OP125799	Bredy15-T22208_ ON448367	Plast15-T22415_ ON448374	Bredy14-T19767_ OP125784	Bredy14-T19736_ OP125782	Bredy14-T19741_ OP125783	Kartaly14-T19346_ OP125793	Kartaly14-T19309_ OP125791	Kartaly14-T19314_ OP125792	Kartaly14-T19658_ OP125795	Fershampenuaz14-T19014_OP125789	Kartaly14-T19551_ OP125794	Bredy15-T22181_ OP125786	Bredy15-T22188_ OP125787	Bredy15-T22189_ OP125788	Kartaly15-T22141_ OP125796	Plast15-T22438_ OP125798	Plast15-T22436_ ON448356
17-L1_MH688537	99.77																											
Erzin14-T20074_MW525323	97.70	97.93																										
XJ-YGTV-1_MT248419	98.62	98.85	98.16																									
YG_MH688530	99.77	100.00	97.93	98.85																								
Republic_Altay/997/2016_MW556731	98.62	98.85	96.76	98.16	98.85																							
Republic_Altay/1001/2016_MW556735	98.62	98.85	96.76	98.16	98.85	100.00																						
Bredy14-T19813_OP125785	98.62	98.85	96.76	97.70	98.85	98.62	98.62																					
Bredy14-T19463_OP125781	98.85	99.08	96.99	97.93	99.08	98.85	98.85	98.39																				
Mir15-T22470_OP125797	98.62	98.85	96.76	97.70	98.85	98.62	98.62	98.16	98.85																			
Zaozernyy15-T22264_OP125799	99.08	99.31	97.23	98.16	99.31	99.08	99.08	98.62	99.31	99.54																		
Bredy15-T22208_ON448367	99.31	99.54	97.46	98.39	99.54	98.85	98.85	98.39	99.08	99.31	99.77																	
Plast15-T22415_ON448374	99.08	99.31	97.23	98.16	99.31	99.08	99.08	98.62	99.31	99.54	100.00	99.77																
Bredy14-T19767_OP125784	99.31	99.54	97.46	98.39	99.54	98.85	98.85	98.39	99.08	99.31	99.77	100.00	99.77															
Bredy14-T19736_OP125782	98.62	98.85	96.76	97.70	98.85	98.62	98.62	98.16	98.85	99.08	99.54	99.31	99.54	99.31														
Bredy14-T19741_OP125783	99.08	99.31	97.23	98.16	99.31	99.08	99.08	98.62	99.31	99.54	100.00	99.77	100.00	99.77	99.54													
Kartaly14-T19346_OP125793	98.16	98.39	96.29	97.70	98.39	98.16	98.16	97.70	98.39	98.62	99.08	98.85	99.08	98.85	98.62	99.08												
Kartaly14-T19309_OP125791	98.85	99.08	96.99	97.93	99.08	98.85	98.85	98.39	99.08	98.85	99.31	99.08	99.31	99.08	98.85	99.31	98.39											
Kartaly14-T19314_OP125792	99.08	99.31	97.23	98.16	99.31	99.08	99.08	98.62	99.77	99.08	99.54	99.31	99.54	99.31	99.08	99.54	98.62	99.31										
Kartaly14-T19658_OP125795	99.31	99.54	97.46	98.39	99.54	99.31	99.31	98.85	99.54	99.31	99.77	99.54	99.77	99.54	99.31	99.77	98.85	99.54	99.77									
Fershampenuaz14-T19014_OP125789	98.39	98.62	96.76	97.46	98.62	98.39	98.39	97.93	99.08	98.85	99.31	99.08	99.31	99.08	98.85	99.31	98.39	98.62	98.85	99.08								
Kartaly14-T19551_OP125794	98.85	99.08	96.99	97.93	99.08	98.85	98.85	98.39	99.08	99.31	99.77	99.54	99.77	99.54	99.31	99.77	98.85	99.08	99.31	99.54	99.08							
Bredy15-T22181_OP125786	98.85	99.08	97.46	98.39	99.08	98.85	98.85	98.39	99.08	99.31	99.77	99.54	99.77	99.54	99.31	99.77	98.85	99.08	99.31	99.54	99.08	99.54						
Bredy15-T22188_OP125787	98.85	99.08	96.99	97.93	99.08	98.85	98.85	98.85	99.08	99.31	99.77	99.54	99.77	99.54	99.31	99.77	98.85	99.08	99.31	99.54	99.08	99.54	99.54					
Bredy15-T22189_OP125788	98.85	99.08	96.99	97.93	99.08	98.85	98.85	98.39	99.54	99.31	99.77	99.54	99.77	99.54	99.31	99.77	98.85	99.08	99.31	99.54	99.54	99.54	99.54	99.54				
Kartaly15-T22141_OP125796	98.39	98.62	96.52	97.46	98.62	98.39	98.39	97.93	98.62	99.31	99.31	99.08	99.31	99.08	99.31	99.31	98.39	98.62	98.85	99.08	98.62	99.08	99.08	99.08	99.08			
Plast15-T22438_OP125798	98.62	98.85	96.76	97.70	98.85	98.62	98.62	98.16	98.85	100.00	99.54	99.31	99.54	99.31	99.08	99.54	98.62	98.85	99.08	99.31	98.85	99.31	99.31	99.31	99.31	99.31		
Plast15-T22436_ON448356	99.08	99.31	97.23	98.16	99.31	99.08	99.08	98.62	99.31	99.54	100.00	99.77	100.00	99.77	99.54	100.00	99.08	99.31	99.54	99.77	99.31	99.77	99.77	99.77	99.77	99.31	99.54	
Gubenka15-T22237_OP125790	99.08	99.31	97.23	98.16	99.31	99.08	99.08	98.62	99.31	99.54	100.00	99.77	100.00	99.77	99.54	100.00	99.08	99.31	99.54	99.77	99.31	99.77	99.77	99.77	99.77	99.31	99.54	100.00

Analyses were conducted using the Poisson correction model [9]. This analysis involved 29 amino acid sequences. The coding data was translated assuming a Standard genetic code table. All positions containing gaps and missing data were eliminated (complete deletion option). There were a total of 439 positions in the final dataset. Evolutionary analyses were

conducted in MEGA X [8]. Colour indicates from what tick species the strain was isolated: green – *Dermacentor nuttalli*, blue – *Dermacentor marginatus*, orange – *Dermacentor reticulatus*, red – *Ixodes persulcatus*.

Table S10. Percent identity of the nucleotide sequences of the protein VP1b of 16 strains of the Yanggou tick virus. GenBank accession numbers for each strain are presented in the table.

Strain_GenBank accession number	16-T2_MH688533	17-L1_MH688537	Erzin14-T20074_MW525323	XJ-YGTV-1_MT248419	YG_MH688530	Republic_Altay/997/2016_MW556731	Republic_Altay/1001/2016_MW556735	Bredy14-T19767_OP125784	Kartaly14-T19309_OP125791	Kartaly14-T19658_OP125795	Fershampenuaz14-T19014_OP125789	Kartaly14-T19551_OP125794	Bredy15-T22181_OP125786	Bredy15-T22188_OP125787	Bredy15-T22189_OP125788
17-L1_MH688537	99.25														
Erzin14-T20074_MW525323	93.58	93.59													
XJ-YGTV-1_MT248419	95.41	95.42	94.19												
YG_MH688530	99.12	98.61	93.43	95.00											
Republic_Altay/997/2016_MW556731	97.71	97.97	93.74	95.43	97.57										
Republic_Altay/1001/2016_MW556735	97.71	97.97	93.74	95.43	97.57	100.00									
Bredy14-T19767_OP125784	96.92	97.45	93.34	95.30	96.53	97.45	97.45								
Kartaly14-T19309_OP125791	96.24	96.51	93.15	94.59	96.11	97.31	97.31	98.10							
Kartaly14-T19658_OP125795	96.24	96.51	93.15	94.59	96.11	97.31	97.31	98.10	100.00						
Fershampenuaz14-T19014_OP125789	96.92	97.44	93.32	95.56	96.78	97.71	97.71	99.00	98.35	98.35					
Kartaly14-T19551_OP125794	96.25	97.04	92.88	94.87	96.11	97.32	97.32	98.61	97.70	97.70	98.87				
Bredy15-T22181_OP125786	96.65	97.18	93.04	95.02	96.52	97.45	97.45	99.25	98.09	98.09	98.99	98.35			
Bredy15-T22188_OP125787	97.05	97.57	93.46	95.43	96.91	97.58	97.58	98.87	98.22	98.22	99.12	98.48	98.87		
Bredy15-T22189_OP125788	96.78	97.31	93.17	95.42	96.64	97.57	97.57	98.87	98.22	98.22	99.62	98.74	98.86	98.99	
Plast15-T22438_OP125798	96.78	97.31	93.45	95.15	96.65	97.31	97.31	98.61	98.22	98.22	98.87	98.22	98.61	99.50	98.74

Analyses were conducted using the Maximum Composite Likelihood model [7]. This analysis involved 16 nucleotide sequences. Codon positions included were 1st+2nd+3rd+Noncoding. All positions containing gaps and missing data were eliminated (complete deletion option). There were a total of 804 positions in the final dataset. Evolutionary analyses were conducted in MEGA X [8]. Colour indicates from what tick species the strain was isolated: green – *Dermacentor nuttalli*, blue – *Dermacentor marginatus*, orange – *Dermacentor reticulatus*.

Table S11. Percent identity of the amino acid sequences of the protein VP1b of 16 strains of the Yanggou tick virus. GenBank accession numbers for each strain are presented in the table.

Strain_GenBank accession number	16-T2_ MH688533	17-L1_ MH688537	Erzin14-T20074_ MW525323	XJ-YGTV-1_ MT248419	YG_ MH688530	Republic_Altay/997/2016_ MW556731	Republic_Altay/1001/2016_ MW556735	Bredy14-T19767_ OP125784	Kartaly14-T19309_ OP125791	Kartaly14-T19658_ OP125795	Fershampenuaz14-T19014_OP125789	Kartaly14-T19551_ OP125794	Bredy15-T22181_ OP125786	Bredy15-T22188_ OP125787	Bredy15-T22189_ OP125788
17-L1_MH688537	99.62														
Erzin14-T20074_MW525323	99.25	99.62													
XJ-YGTV-1_MT248419	98.49	98.87	99.25												
YG_MH688530	99.62	100.00	99.62	98.87											
Republic_Altay/997/2016_MW556731	99.25	99.62	100.00	99.25	99.62										
Republic_Altay/1001/2016_MW556735	99.25	99.62	100.00	99.25	99.62	100.00									
Bredy14-T19767_OP125784	99.25	99.62	99.25	98.49	99.62	99.25	99.25								
Kartaly14-T19309_OP125791	99.25	99.62	100.00	99.25	99.62	100.00	100.00	99.25							
Kartaly14-T19658_OP125795	99.25	99.62	100.00	99.25	99.62	100.00	100.00	99.25	100.00						
Fershampenuaz14-T19014_OP125789	99.62	100.00	99.62	98.87	100.00	99.62	99.62	99.62	99.62	99.62					
Kartaly14-T19551_OP125794	98.87	99.25	98.87	98.11	99.25	98.87	98.87	99.62	98.87	98.87	99.25				
Bredy15-T22181_OP125786	99.62	100.00	99.62	98.87	100.00	99.62	99.62	99.62	99.62	99.62	100.00	99.25			
Bredy15-T22188_OP125787	99.25	99.62	99.25	98.49	99.62	99.25	99.25	99.25	99.25	99.25	99.62	98.87	99.62		
Bredy15-T22189_OP125788	99.25	99.62	99.25	98.49	99.62	99.25	99.25	99.25	99.25	99.25	99.62	98.87	99.62	99.25	
Plast15-T22438_OP125798	99.25	99.62	99.25	98.49	99.62	99.25	99.25	99.25	99.25	99.25	99.62	98.87	99.62	100.00	99.25

Analyses were conducted using the Poisson correction model [9]. This analysis involved 16 amino acid sequences. The coding data was translated assuming a Standard genetic code table. All positions containing gaps and missing data were eliminated (complete deletion option). There were a total of 267 positions in the final dataset. Evolutionary analyses were conducted in MEGA X [8]. Colour indicates from what tick species the strain was isolated: green – *Dermacentor nuttalli*, blue – *Dermacentor marginatus*, orange – *Dermacentor reticulatus*.

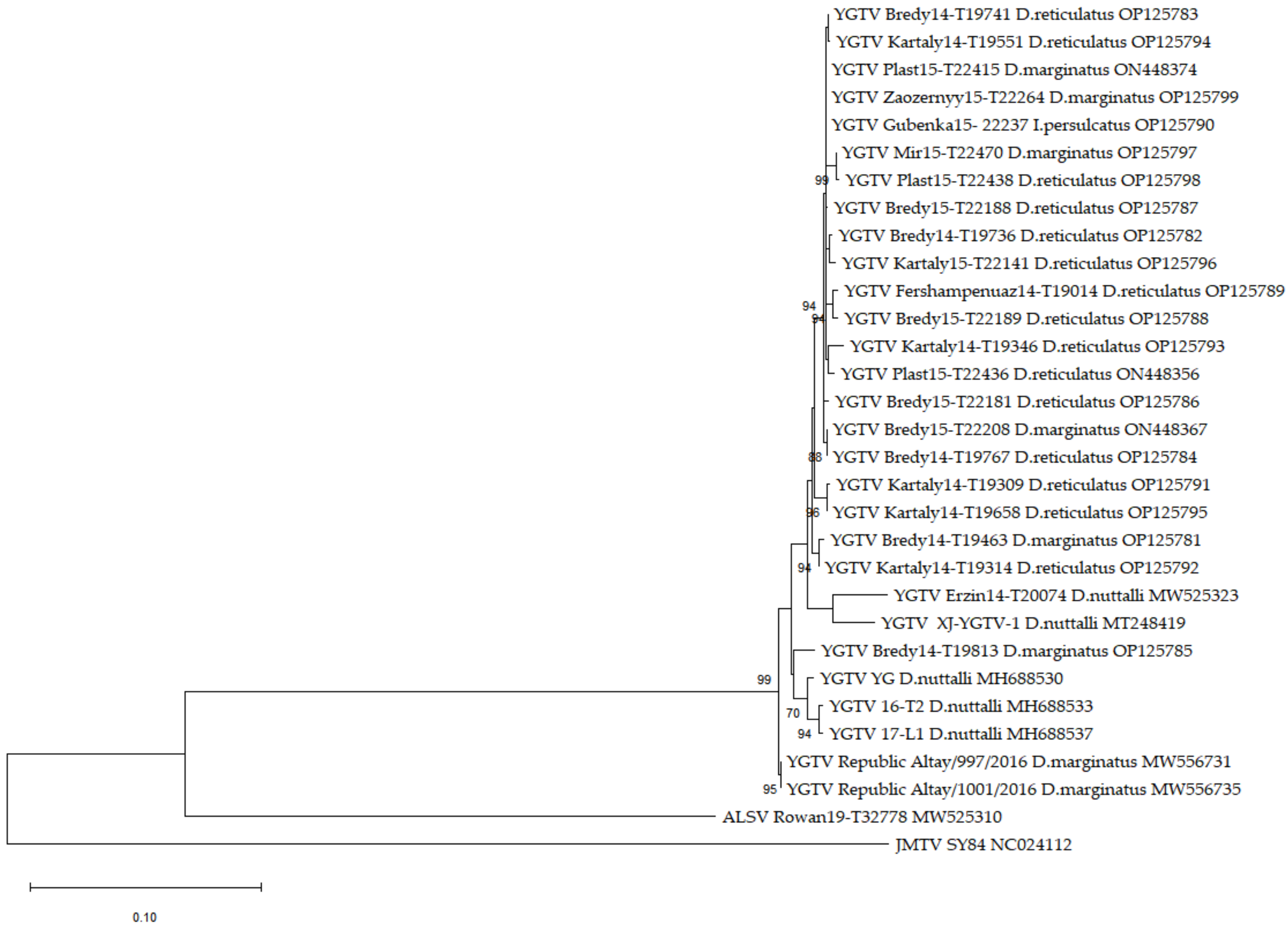

Figure S1. Phylogenetic analysis of the complete nucleotide sequence of the Yanggou tick virus VP1a protein. Phylogenetic tree was constructed in MEGA X with the Maximum Likelihood method (1000 bootstrap replications). Bootstrap values (> 70%) are shown at the branches. GenBank accession numbers are listed for each enteries. Yanggou tick virus = YGTV, Alongshan virus = ALSV, Jingmen tick virus = JMTV.

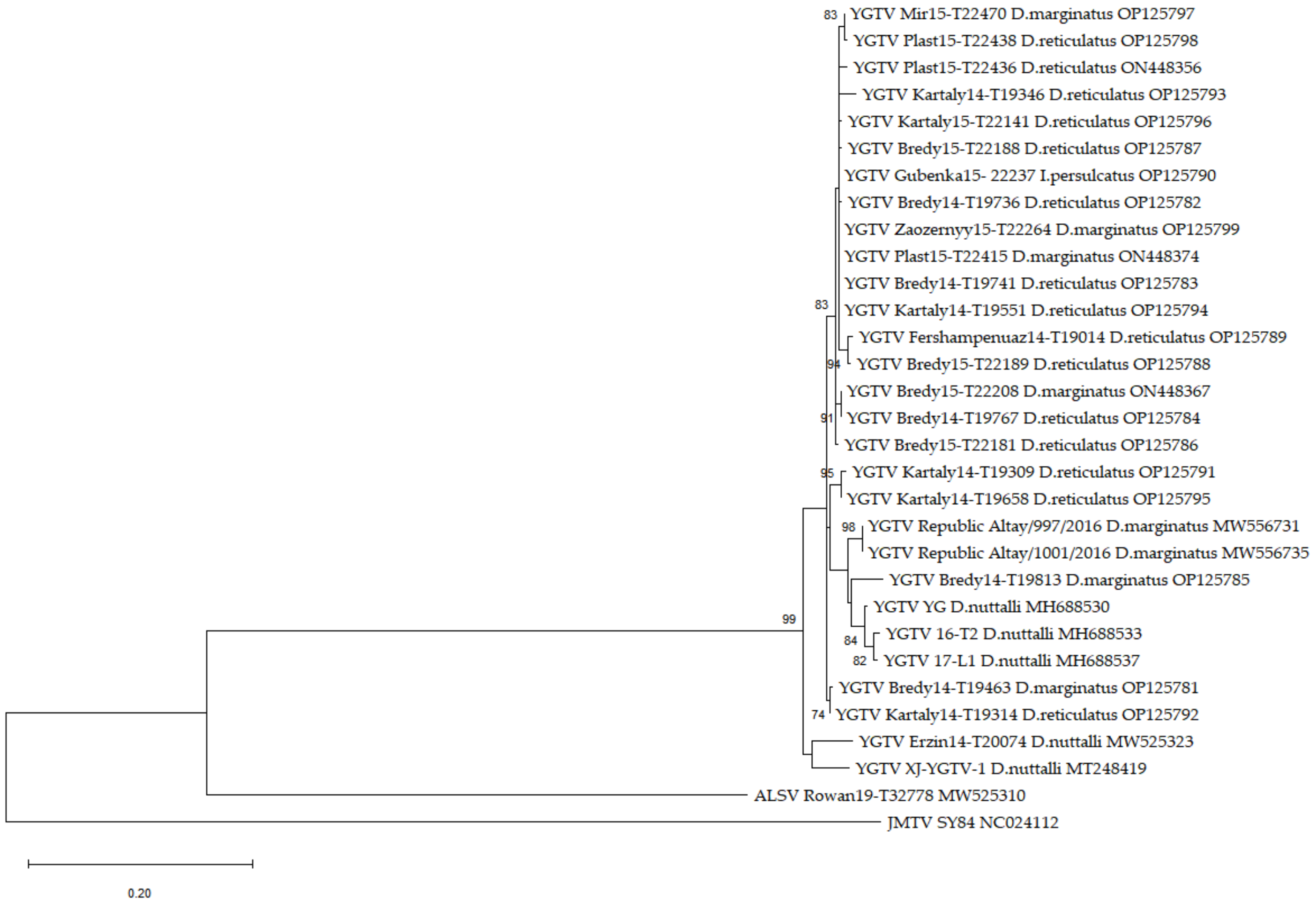

Figure S2. Phylogenetic analysis of the complete amino acid sequence of the Yanggou tick virus VP1a protein. Phylogenetic tree was constructed in MEGA X with the Maximum Likelihood method (1000 bootstrap replications). Bootstrap values (> 70%) are shown at the branches. GenBank accession numbers are listed for each enteries. Yanggou tick virus = YGTV, Alongshan virus = ALSV, Jingmen tick virus = JMTV.

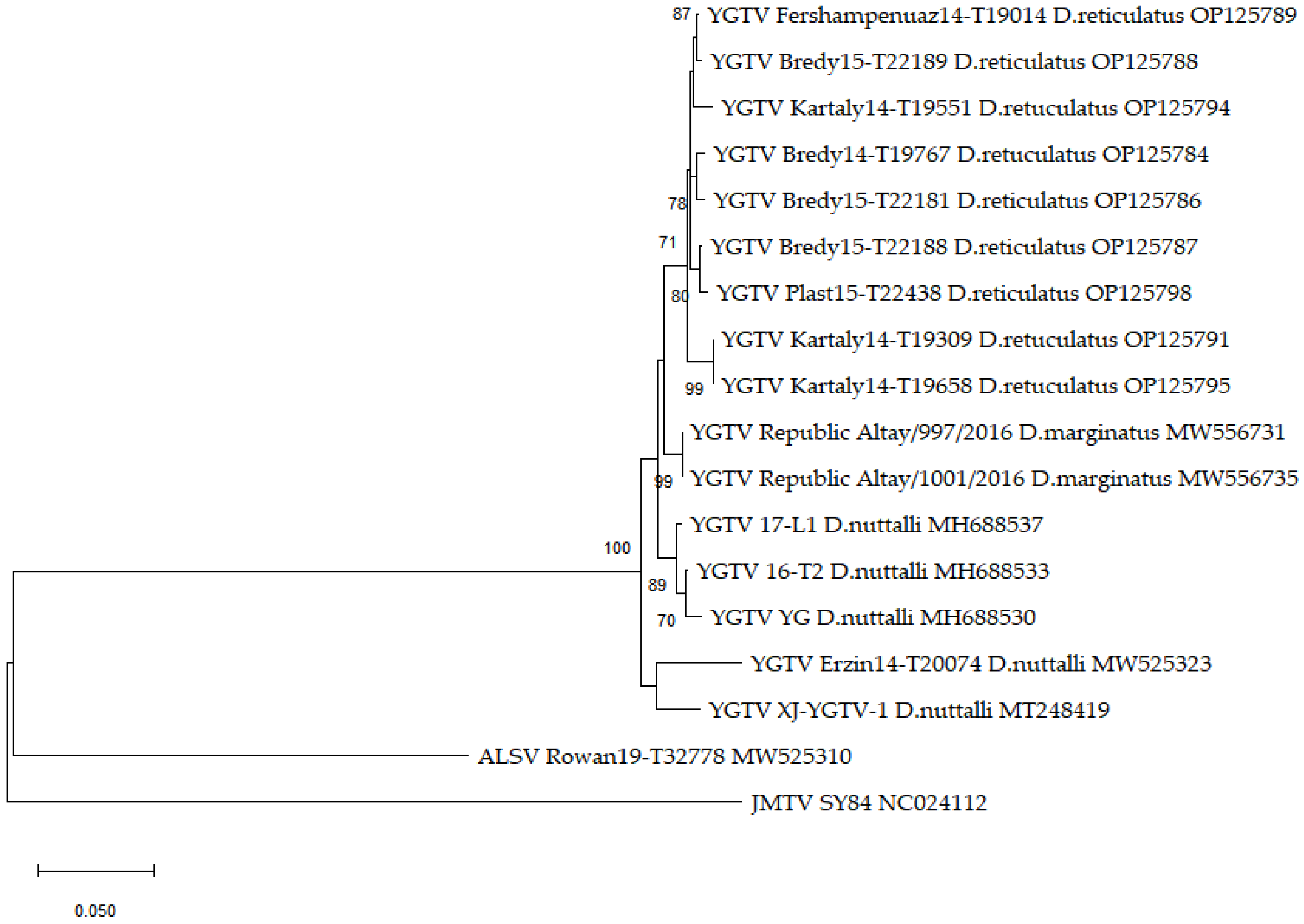

Figure S3. Phylogenetic analysis of the complete nucleotide sequence of the Yanggou tick virus VP1b protein. Phylogenetic tree was constructed in MEGA X with the Maximum Likelihood method (1000 bootstrap replications). Bootstrap values (> 70%) are shown at the branches. GenBank accession numbers are listed for each enteries. Yanggou tick virus = YGTV, Alongshan virus = ALSV, Jingmen tick virus = JMTV.

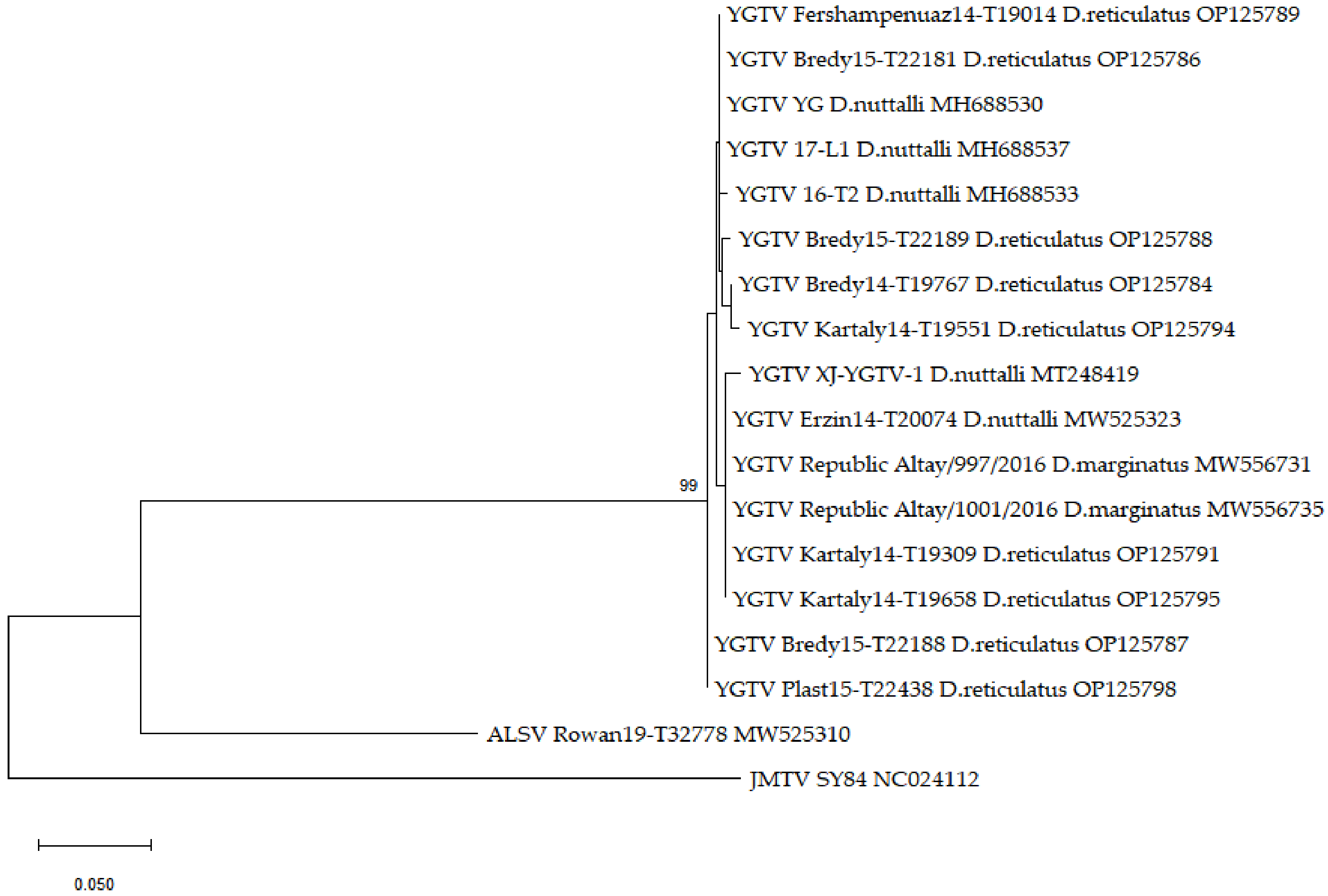

Figure S4. Phylogenetic analysis of the complete amino acid sequence of the Yanggou tick virus VP1b protein. Phylogenetic tree was constructed in MEGA X with the Maximum Likelihood method (1000 bootstrap replications). Bootstrap values (> 70%) are shown at the branches. GenBank accession numbers are listed for each enteries. Yanggou tick virus = YGTV, Alongshan virus = ALSV, Jingmen tick virus = JMTV.

References

1. Kholodilov, I.S.; Belova, O.A.; Morozkin, E.S.; Litov, A.G.; Ivannikova, A.Y.; Makenov, M.T.; Shchetinin, A.M.; Aibulatov, S. V.; Bazarova, G.K.; Bell-Sakyi, L.; et al. Geographical and Tick-Dependent Distribution of Flavi-like Alongshan and Yanggou Tick Viruses in Russia. *Viruses* **2021**, *13*, 458, doi:10.3390/v13030458.

2. Kholodilov, I.S.; Litov, A.G.; Klimentov, A.S.; Belova, O.A.; Polienko, A.E.; Nikitin, N.A.; Shchetinin, A.M.; Ivannikova, A.Y.; Bell-Sakyi, L.; Yakovlev, A.S.; et al. Isolation and characterisation of Alongshan virus in Russia. *Viruses* **2020**, *12*, 362, doi:10.3390/v12040362.

3. Romanova, L.I.; Kozlovskaya, L.I.; Shevtsova, A.S.; Karganova, G.G. Evidence for the absence of tick-borne encephalitis virus RNA in bioassays. *Vopr. Virusol.* **2006**, *51*, 38–41.

4. Kholodilov, I.; Belova, O.; Burenkova, L.; Korotkov, Y.; Romanova, L.; Morozova, L.; Kudriavtsev, V.; Gmyl, L.; Belyaletdinova, I.; Chumakov, A.; et al. Ixodid ticks and tick-borne encephalitis virus prevalence in the South Asian part of Russia (Republic of Tuva). *Ticks Tick. Borne. Dis.* **2019**, *10*, 959–969, doi:10.1016/j.ttbdis.2019.04.019.

5. Scaramozzino, N.; Crance, J.-M.; Jouan, A.; DeBriel, D.A.; Stoll, F.; Garin, D. Comparison of Flavivirus

universal primer pairs and development of a rapid, highly sensitive heminested reverse transcription-PCR assay for detection of flaviviruses targeted to a conserved region of the NS5 gene sequences. *J. Clin. Microbiol.* **2001**, *39*, 1922–1927, doi:10.1128/JCM.39.5.1922-1927.2001.

6. Klimentov, A.S.; Butenko, A.M.; Khutoretskaya, N. V.; Shustova, E.Y.; Larichev, V.F.; Isaeva, O. V.; Karganova, G.G.; Lukashev, A.N.; Gmyl, A.P. Development of pan-phlebovirus RT-PCR assay. *J. Virol. Methods* **2016**, *232*, 29–32, doi:10.1016/j.jviromet.2016.02.009.

7. Tamura, K.; Nei, M.; Kumar, S. Prospects for inferring very large phylogenies by using the neighbor-joining method. *Proc. Natl. Acad. Sci. U. S. A.* **2004**, *101*, 11030–11035, doi:10.1073/pnas.0404206101.

8. Kumar, S.; Stecher, G.; Li, M.; Knyaz, C.; Tamura, K. MEGA X: Molecular evolutionary genetics analysis across computing platforms. *Mol. Biol. Evol.* **2018**, *35*, 1547–1549, doi:10.1093/molbev/msy096.

9. Zuckerkandl, E.; Pauling, L. Evolutionary divergence and convergence in proteins. In *Evolving Genes and Proteins*; Bryson, V., Vogel, H.J., Eds.; Academic Press: New York and London, 1965; pp. 97–166.
